# Risk of neurologic or immune-mediated adverse events after COVID-19 diagnosis in the United States

**DOI:** 10.1371/journal.pone.0333704

**Published:** 2025-11-24

**Authors:** Shelby S. Fisher, Arnstein Lindaas, Stella G. Muthuri, Patricia C. Lloyd, Joann F. Gruber, Morgan M. Richey, Hai Lyu, Angela S. Cheng, Lisa S. Kowarski, Mollie M. McKillop, Christine Bui, Tainya C. Clarke, Jeffrey Beers, Timothy Burrell, Pablo Freyria Duenas, Yangping Chen, Minya Sheng, Richard A. Forshee, Steven A. Anderson, Yoganand Chillarige, Mary S. Anthony, Azadeh Shoaibi, J. Bradley Layton

**Affiliations:** 1 RTI International, Durham, North Carolina, United States of America; 2 Acumen LLC, Burlingame, California, United States of America; 3 IBM Consulting, Bethesda, Maryland, United States of America; 4 United States of America Food and Drug Administration, Center for Biologics Evaluation and Research, Silver Spring, Maryland, United States of America; 5 RTI Health Solutions, Durham, North Carolina, United States of America; Taipei Medical University, TAIWAN

## Abstract

**Introduction:**

Neurologic or immune-mediated conditions have been evaluated as potential adverse events (AEs) in coronavirus disease 2019 (COVID-19) vaccine safety surveillance. To contextualize United States (US) surveillance findings, it is important to quantify the association of AEs with COVID-19 diagnoses among US adults before the introduction of COVID-19 vaccines.

**Methods:**

Cohort and self-controlled risk interval (SCRI) designs were used in 2 US administrative claims data sources—Merative™ MarketScan^®^ Commercial Database (ages 18−64 years) and Medicare fee-for-service data (ages ≥ 65 years). AEs included Guillain-Barré syndrome (GBS), Bell’s palsy, encephalitis/encephalomyelitis, narcolepsy, immune thrombocytopenia (ITP), and transverse myelitis. The cohort (study period, 1 April 2020−10 December 2020) included adults with COVID-19 diagnoses and matched comparators. Inverse probability of treatment-weighted hazard ratios (HRs) and 95% confidence intervals (CIs) were estimated. The SCRI (study period, 1 June 2020−10 December 2020) identified the AEs in risk windows after COVID-19 diagnosis and pre- and postexposure reference windows. Relative incidences (RIs) and 95% CIs were estimated with seasonality-adjusted conditional Poisson regression models accounting for outcome-dependent observation windows.

**Results:**

The study observed a consistent association between COVID-19 diagnosis and GBS: MarketScan HR = 9.57 (95% CI, 1.23–74.74), RI = 8.53 (95% CI, 2.45–29.7); Medicare HR = 1.97 (95% CI, 1.04–3.74), RI = 4.63 (95% CI, 1.78–12.01). For ITP, the association was weaker, but still consistently elevated: MarketScan HR = 2.06 (95% CI, 1.20–3.53), RI = 1.74 (95% CI, 1.01–3.00); Medicare HR = 1.36 (95% CI, 1.18–1.57), RI = 1.91 (95% CI, 1.60–2.28). For all remaining AEs, there was not consistent evidence of an association with COVID-19, with estimates that were generally modest, imprecise, or varying by study design.

**Conclusions:**

COVID-19 diagnoses were associated with an increased risk of GBS and ITP in both data sources and study designs. Increased risks of other neurologic/immune-mediated AEs cannot be ruled out.

## Introduction

The coronavirus disease 2019 (COVID-19) pandemic resulted in substantial morbidity and mortality in the United States (US) and around the world. Infection with the SARS-CoV-2 virus ranges in severity from asymptomatic to severe and potentially fatal, and varying perceptions of the seriousness of the disease continue. Understanding the risk of adverse events (AEs) after a diagnosis of COVID-19 is important both for clinical care after COVID-19 and for better understanding the benefit-risk profile of vaccines relative to risks associated with the disease, providing important context for vaccine safety surveillance findings.

Understanding COVID-19’s association with neurological and immune-mediated conditions has been of particular interest given observed direct tissue damage, pro-inflammatory response, and downstream negative effects of hyperinflammation on the nervous system following COVID-19 [1–4]. Initially jumpstarted as a response to a SARS-CoV-2 infection, the immune system can also mount a dysregulated response that leads to a variety of potential immune issues: marked cytokine release syndrome, systemic hyperinflammation, hyperactivation of T cells, and the release of high levels of pro-inflammatory cytokines (e.g., IL-1β, IL-6, TNFα), in other words, “a cytokine storm.” Such responses have been linked to inflammatory, autoimmune, or neurological conditions, including Guillain-Barré syndrome (GBS), Bell’s palsy, encephalitis/encephalomyelitis, narcolepsy, immune thrombocytopenia (ITP), and transverse myelitis [5–13]. Previous studies from several countries have evaluated the association between various definitions of exposure to SARS-CoV-2 (e.g., COVID-19 hospitalization, diagnosis, or positive test) and select AEs using cohort [14–17], case-control [18], and self-controlled [16,19–21] study designs.

The US Food and Drug Administration (FDA) Center for Biologics Evaluation and Research (CBER) uses the Biologics Effectiveness and Safety (BEST) Initiative to conduct active postmarketing safety surveillance of biologics including vaccines. Using the BEST Initiative, this study utilized 2 large US data sources and 2 study designs (cohort and self-controlled risk interval [SCRI]) to assess the incidence of neurologic or immune-mediated AEs following COVID-19 diagnosis before the introduction of COVID-19 vaccines. All AEs included in this study are those commonly evaluated during vaccine safety studies in US adults.

## Materials and methods

### Data sources

All analyses were performed separately according to a common protocol [22] using 2 administrative health insurance claims databases that were participating in the FDA CBER BEST Initiative: the Merative™ MarketScan^®^ Commercial Database and data from the US Centers for Medicare & Medicaid Services fee-for-service Medicare claims. MarketScan contains insurance claims and enrollment data for employees, spouses, and dependents with employer-based commercial insurance from large employers, health plans, and government and public organizations throughout the US. Medicare is a federal program providing health insurance coverage to individuals aged 65 years and older and individuals aged younger than 65 years who have end-stage renal disease or are disabled. The analyses in MarketScan included individuals between 18 and 64 years old, whereas the Medicare analyses included beneficiaries 65 years of age and older with Part A (inpatient hospital care) and Part B (outpatient care and physician services) coverage. *International Classification of Diseases, Tenth Revision, Clinical Modification* (ICD-10-CM) diagnosis codes were used to identify diagnoses. Current Procedural Terminology, Healthcare Common Procedure Coding System, and ICD-10 *Procedure Coding System* (ICD-10-PCS) codes were used to identify and assess procedures and administered vaccines. In MarketScan, National Drug Codes were also used to identify pharmacy dispensed vaccines (pharmacy information was not required for Medicare). Death information was available only in Medicare. Diagnosis-Related Group codes were also used to identify diagnoses and procedures. In both data sources, an absence of recorded codes for a condition was interpreted as the condition not being present; categorical variables (i.e., geographic region, race/ethnicity) were allowed to take a value of “unknown” if recorded as such in the data source.

The MarketScan data were accessed for research purposes between 22 February 2023 and 8 August 2023; the analyses in Medicare data were conducted between 15 February 2023 and 8 September 2023. This surveillance activity was conducted as part of the FDA public health surveillance mandate. As a public health surveillance activity conducted under the direction of a US public health authority, this activity did not constitute research and was not subject to Institutional Review Board oversight according to US regulations 45 CFR Part 46.102(k) and 46.102(l)(2). Individual-level consent was not required for this analysis of secondary healthcare data. Individual participants could not be identified during or after data collection by the study authors.

### Study design

Two study designs were employed—a cohort and an SCRI design—to account for the unique challenges presented by the pandemic and to capitalize on the strengths and weaknesses of each design. The cohort study (using data from 1 April 2020−10 December 2020) assigned exposure status based on the presence or absence of a recorded COVID-19 diagnosis, and a diagnosed individual was matched to a comparator who was not diagnosed on or before the same calendar date. The SCRI design (using data from 1 June 2020−10 December 2020) included individuals who were diagnosed with COVID-19 and who experienced an AE by comparing the risk of AEs after the COVID-19 diagnosis to reference windows within the same individual. The SCRI design’s use of participants as their own comparators minimized the concerns in the matched cohorts over misclassification of COVID-19 disease (e.g., undiagnosed or false-negative COVID-19 cases in the comparator group) and time-invariant confounding factors that may be difficult to account for (e.g., adherence to preventive measure, risk tolerance, behavioral, or lifestyle factors). However, in contrast to the cohort design, the SCRI analysis required the following assumptions that may have been violated: AE event rates largely remain constant over time; the exposure is transient and identifiable; a biologically relevant risk window for an AE can be clearly defined relative to the COVID-19 diagnosis; and the occurrence of an outcome does not affect the duration of the risk or reference observation windows (e.g., by increasing mortality risk).

Individuals were excluded if they were not continuously enrolled in a contributing insurance plan for at least 1 year before COVID-19 diagnosis (cohort and SCRI) or the occurrence of the AE (SCRI), had no observed healthcare encounters in the year before the COVID-19 diagnosis (cohort only), or had a recorded COVID-19 diagnosis or select respiratory infections before Time 0. AE-specific exclusion criteria were also applied, excluding those with a history of the AE, resulting in a unique analytic data set for each AE in each design, which was used to estimate the association between COVID-19 diagnosis and each AE.

### Variables

Complete details of all variable definitions are given in the publicly available study protocol [22]. Code lists for key variables are given in S1 Table.

### Exposure

In both study designs, the exposure of interest was a COVID-19 diagnosis (ICD-10-CM code U07.1) identified in claims data from inpatient, emergency department, outpatient, or professional/provider entities. In Medicare, codes from all diagnostic positions except the admitting diagnosis code were included because admission codes can be preliminary. The COVID-19 diagnosis date was defined as the relevant service date in each data source (e.g., the admission date for facility-level diagnoses, the service date for provider-level diagnoses). The date of the COVID-19 diagnosis was used as Time 0; in the cohort study, the calendar date of matching became Time 0 for the comparator group.

### Outcomes

**Fig 1:**
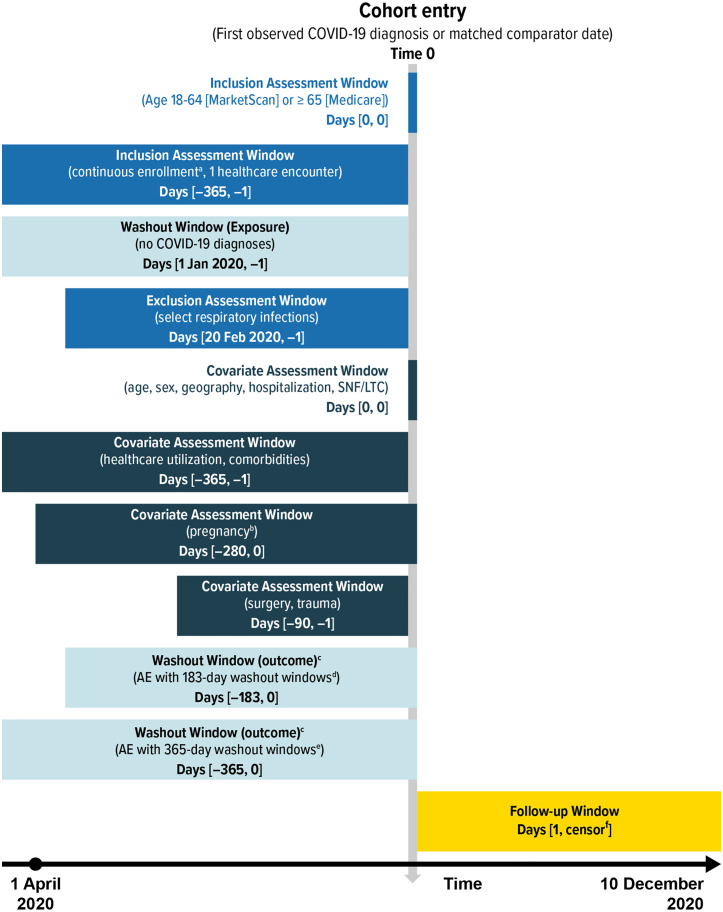
Eligibility assessment, covariate assessment, and follow-up windows relative to time 0 for the cohort design. AE = adverse event; COVID-19 = coronavirus disease 2019; LTC = long-term care; SNF = skilled nursing facility. ^a^ Gaps in insurance coverage of a maximum of 31 days were permitted in MarketScan. ^b^ Pregnancy status identified in MarketScan only. ^c^ Outcome-specific washout windows were applied only to analyses of individual AEs, not to the base study cohort. ^d^ Bell’s palsy and encephalitis/encephalomyelitis. ^e^ All outcomes other than Bell’s palsy and encephalitis/encephalomyelitis. ^f^ Occurrence of the event of interest or censoring at the earliest of the following: disenrollment from the database (gaps in insurance coverage of a maximum of 31 days were permitted in MarketScan); death (available in Medicare only); end of the study period (10 December 2020); or the day before COVID-19 diagnosis in comparators for both the comparator and the individual with COVID-19 to whom they were matched. Follow-up for the cohort design was not censored at the end of the 41-day risk window as was done for the self-controlled risk interval design. Note: The cohort entry period (the time during which all Time 0 dates must occur) began on 1 April 2020, but lookback periods may have extended before 1 April 2020, as far back as 2 April 2019. Note: This figure displays the analyses with follow-up starting on the day after Time 0, and Time 0 included in the washout windows. Additional analyses were performed with follow-up beginning on Time 0, and the washout windows ending on the day before Time 0.

**Fig 2:**
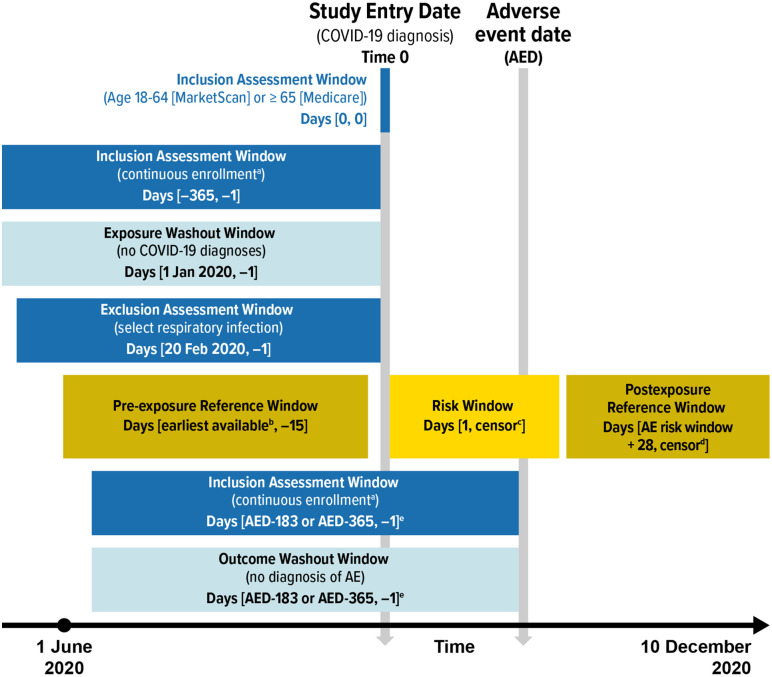
Eligibility Assessment, Covariate Assessment, Risk Windows, and Reference Windows Relative to Time 0 for the Self-Controlled Risk Interval Design. AE = adverse event; AED = adverse event date; COVID-19 = coronavirus disease 2019. ^a^ Gaps of up to 31 days were permitted in MarketScan. ^b^ Latest of the following: 365 days after the beginning of continuous enrollment; beginning of the study period (1 June 2020). ^c^ Earliest of the following: 41 days; disenrollment from the database (gaps in insurance coverage of a maximum of 31 days were permitted); death (available in Medicare only); end of the study period (10 December 2020). ^d^ Earliest of the following: 365 days; disenrollment from the database (gaps insurance coverage of a maximum of 31 days were permitted); death (available in Medicare only); end of the study period (10 December 2020). ^e^ Length of outcome-specific washout window varied by outcome. Note: Covariates for descriptive purposes were evaluated relative to Time 0 using the same process and assessment windows used for the cohort design. Note: The study entry period (the calendar time during which all Time 0 dates must have occurred) began on 1 June 2020. However, lookback periods may have extended before 1 June 2020. Note: This figure displays the analyses with the risk window starting on the day after Time 0. Additional analyses were performed with the risk window beginning on Time 0.

The outcomes for this study included GBS, Bell’s palsy, encephalitis/encephalomyelitis, narcolepsy, ITP, and transverse myelitis; these outcomes, along with their associated risk windows and washout windows (Table 1) have previously been included in COVID-19 vaccine safety surveillance activities [23–26], identified using ICD-10-CM diagnosis codes (S1 and S2 Tables). In the cohort, outcomes were identified during all-available follow-up time; in the SCRI, outcomes were identified during 41-day risk windows after the COVID-19 diagnosis and in pre-exposure or postexposure reference windows. AE-specific washout windows were employed in both study designs to exclude those with histories of the AE to ensure that AEs identified during follow-up were incident events rather than recorded diagnoses for continuing care of previously occurring AEs (Table 1). Depending on the nature of each AE (e.g., acute or chronic condition), either 6- or 12-month washout windows were applied, consistent with how these AEs have been operationalized in COVID-19 vaccine surveillance activities [23,24]. For the cohort analysis, the outcome washout windows were before or on the date of COVID-19 diagnosis (Time 0) or the matched index date for comparators (Fig 1), and for the SCRI analysis, the outcome washout windows were before the date of the AEs (Fig 2).

**Table 1:** Analysis set for each outcome and definition.

Adverse event	Risk window for SCRI^a^	Washout window^b^
Guillain-Barré syndrome	Day 1–41	365 days
Bell’s palsy	Day 1–41	183 days
Encephalitis/encephalomyelitis	Day 1–41	183 days
Narcolepsy	Day 1–41	365 days
Immune thrombocytopenia	Day 1–41	365 days
Transverse myelitis	Day 1–41	365 days

AE = adverse event; COVID-19 = coronavirus disease 2019; SCRI = self-controlled risk interval.

^a^For the SCRI analysis, the risk window was the 41 days after the date of the COVID-19 diagnosis (i.e., Time 0). Additional analyses were also performed including Time 0 in the risk window.

^b^For the cohort analysis, the washout windows is applied to the specified period before and including the date of COVID-19 diagnosis or matched date (i.e., Time 0). For the SCRI, the washout window is applied to the specified period before and not including the date of the AE occurring in the risk or reference window.

### Covariates

Covariates were defined using uniform definitions across both data sources and included demographic and geographic characteristics, comorbidities, and healthcare utilization. Covariates were used to describe the characteristics of each study sample and to estimate propensity scores in the cohort analysis (S3 Table).

### Follow-up

The study protocol [22] prespecified primary analyses that began follow-up or risk windows on Time 0 (i.e., the date of COVID-19 diagnosis or matched calendar date) for the cohort and SCRI analyses; the protocol also prespecified secondary, alternative risk period analyses starting follow-up/risk windows on the day after Time 0. However, given selection bias and potential reverse causality observed in the analyses including Time 0 in follow-up/risk windows (described further in the discussion), this manuscript focuses on the analyses starting follow-up/risk windows on the day after Time 0; those starting on Time 0 are presented as supplementary findings.

### Cohort study statistical methods

The cohort design compared the hazard of AEs in individuals with COVID-19 diagnoses and matched comparators without a COVID-19 diagnosis (Fig 1). The study period for the cohort analyses spanned the period between the date the ICD-10-CM code for COVID-19 (U07.1) was introduced (1 April 2020) and the day before the first COVID-19 vaccine was authorized for use in the US (10 December 2020). This period removed the potential for vaccine-associated AEs being counted as COVID-19-associated AEs. Individuals were identified at their first COVID-19 diagnosis during the study period; the diagnosis date was used as Time 0. On each calendar date of the study period, individuals in the COVID-19-diagnosed group were 1:1 exact matched with replacement to comparators on age (5-year increments), sex, US county (Medicare) or metropolitan statistical area (MarketScan), immunocompromised status, hospitalization status on Time 0, and skilled nursing facility/long-term care residence on Time 0 (Table 2). Comparators who were matched to an individual in the COVID-19-diagnosed group remained eligible to enter the exposed group (i.e., be diagnosed with COVID-19) or act as a comparator to an additional individual in the COVID-19 group as long as they remained eligible at a given Time 0.

**Table 2:** Characteristics of included individuals for SCRI and cohort analyses.

Characteristic	SCRI design		Cohort design			
	MarketScan	Medicare	MarketScan		Medicare	
			Exposure group (COVID-19)	Comparator group	Exposure group (COVID-19)	Comparator group
Total observations^ a^ (unique individuals)	330,799	855,065	319,300 (319,300)	319,300 (312,282)	1,017,410 (1,017,410)	1,017,410 (930,513)
Age, years						
Mean (SD)	41.4 (13.7)	77.4 (8.4)	41.8 (13.6)	41.8 (13.6)	77.7 (8.6)	77.7 (8.6)
Median (Q1, Q3)	42 (29, 53)	76 (70, 83)	43 (30, 53)	43 (30, 53)	76 (71, 84)	76 (71, 84)
Sex						
Female	180,800 (54.7)	486,381 (56.9)	178,975 (56.05)	178,975 (56.05)	590,584 (58.0)	590,584 (58.0)
Male	149,999 (45.3)	368,684 (43.1)	140,325 (43.95)	140,325 (43.95)	426,826 (42.0)	426,826 (42.0)
US geographic region, n (%)						
Northeast	44,345 (13.4)	125,327 (14.7)	62,215 (19.48)	62,224 (19.49)	218,277 (21.5)	218,107 (21.4)
North central	69,166 (20.9)	233,154 (27.3)	71,170 (22.29)	71,149 (22.28)	250,139 (24.6)	250,130 (24.6)
South	172,142 (52.0)	355,853 (41.6)	149,868 (46.94)	149,880 (46.94)	385,012 (37.8)	385,108 (37.9)
West	44,841 (13.6)	138,058 (16.1)	36,037 (11.29)	36,037 (11.29)	161,002 (15.8)	161,085 (15.8)
Unknown	305 (0.1)	2,673 (0.3)	10 (0.00)	10 (0.00)	2,980 (0.3)	2,980 (0.3)
Race/ethnicity, n (%)^ b^						
American Indian/Alaska Native	NA	7,165 (0.8)	NA	NA	7,510 (0.7)	5,240 (0.5)
Asian/Pacific Islander	NA	12,648 (1.5)	NA	NA	16,819 (1.7)	19,101 (1.9)
Black (or African American)	NA	67,441 (7.9)	NA	NA	90,997 (8.9)	87,757 (8.6)
Hispanic	NA	21,394 (2.5)	NA	NA	26,460 (2.6)	16,390 (1.6)
Non-Hispanic White	NA	722,056 (84.4)	NA	NA	845,042 (83.1)	857,204 (84.3)
Other	NA	10,840 (1.3)	NA	NA	13,898 (1.4)	14,649 (1.4)
Unknown	NA	13,521 (1.6)	NA	NA	16,684 (1.6)	17,069 (1.7)
Hospitalized on Time 0, n (%)	10,129 (3.1)	182,913 (21.4)	4,046 (1.27)	4,046 (1.27)	166,154 (16.3)	166,154 (16.3)
SNF/LTC ^b^ residence on Time 0, n (%)	NA	186,367 (21.8)	NA	NA	250,193 (24.6)	250,193 (24.6)
Immunocompromised state, n (%)	35,924 (10.9)	357,587 (41.8)	37,672 (11.80)	37,672 (11.80)	438,954 (43.1)	438,954 (43.1)

COVID-19 = coronavirus disease 2019; LTC = long-term care; NA = not applicable; Q1 = first quartile; Q3 = third quartile; SD = standard deviation; SCRI = self-controlled risk interval; SNF = skilled nursing facility; US = United States.

^a^A unique individual may have been included multiple times because of matching with replacement, and an individual may have been included in both the exposure and comparator groups. This table counts each instance of an individual’s entry separately as distinct observations; the number of unique individuals is reported separately.

^b^Data on race/ethnicity and SNF/LTC residence are only available in Medicare.

After matching but before the application of outcome-specific exclusion criteria, patient characteristics were described in the overall matched cohort and absolute standard difference (ASD) values were calculated for each AE to assess covariate balance between the COVID-19-diagnosed group and matched comparators [27]. For each AE, after outcome-specific exclusion requirements were applied, an AE-specific propensity score model was estimated using a priori specified covariates (S3 Table), including potential confounders for each AE [28,29]. The distributions of propensity scores were evaluated by exposure groups. The propensity scores were utilized to estimate stabilized inverse probability of treatment (sIPT) weights for each AE analysis. sIPT weights were truncated below the 1st and above the 99th percentiles to minimize the impact of extreme weights.

Individuals were followed from the day after Time 0 until the occurrence of an AE or censoring at the end of the study period (10 December 2020), disenrollment from the database, death (Medicare, only), or the day before COVID-19 diagnosis in the matched comparator for both individuals in the matched pair (Fig 1). Additional analyses were also conducted in which follow-up began on Time 0. In the sIPT-weighted cohorts, Kaplan-Meier estimates of survival were generated [30] with robust variance estimators to estimate 95% CIs accounting for reuse of comparators [31]. The daily cumulative incidence estimates were calculated for each follow-up day by subtracting the Kaplan-Meier survival estimate and 95% CI from 1. Cox proportional hazard models were used to estimate sIPT-weighted hazard ratios (HRs), and 95% CIs were estimated using robust sandwich variance estimators.

### SCRI statistical methods

Disruptions in healthcare utilization during the early months of the pandemic resulted in a reduced number of diagnoses for a variety of conditions [32–34], potentially violating an assumption of the SCRI analysis that outcomes are consistent over the time period [35]. The SCRI study period thus began on 1 June 2020, when incidence rates of many AEs returned to pre-pandemic levels in US administrative healthcare claims databases [32,34], and ended 10 December 2020. The SCRI design [35] included those diagnosed with COVID-19 and AEs, and compared an individual’s risk of AEs during the post-COVID-19 risk window compared to pre-exposure and postexposure reference windows (Fig 2). Individuals included in the SCRI analyses were followed from the day after Time 0 until disenrollment from the database, death, or the end of the study period (10 December 2020) (Fig 2). Additional analyses were also included that began the risk window on Time 0, the date of COVID-19 diagnosis. Pre-exposure reference windows were defined as the earliest date data were available for a patient during the SCRI study period up until 15 days before COVID-19 diagnosis; the postexposure reference windows were defined as 28 days after the AE risk window until death, disenrollment, or the end of the SCRI study period (Fig 2). Because of the relatively short SCRI study period, the lengths of the reference windows were allowed to vary (i.e., individual’s follow-up could be censored during the windows) to avoid the overly strict requirement that individuals be continuously enrolled throughout a fixed postexposure reference window). Buffer periods, which occurred immediately before and after the risk window, were not included in either risk or reference windows. Although individuals’ follow-up could be censored during the risk or postexposure reference window, they were required to have at least 1 day in the pre-exposure reference window to be included.

Conditional Poisson regression models were used to assess the relative incidence (RI) and 95% CI of each AE and a COVID-19 diagnosis [36,37], accounting for differences in follow-up time in the risk and reference windows; models accounted for seasonal trends in AEs by adjusting for calendar months. Because some outcomes may increase the risk of mortality, the assumption of having no event-dependent censoring may have been violated [38]; thus, an extended Poisson model accounting for event-dependent observation windows was used [39]. Encephalitis/encephalomyelitis was not evaluated with the SCRI design because of its known high case fatality rate. To contextualize the RI observed in the SCRI analysis, attributable risk (the number of excess cases of the AE observed per 100,000 COVID-19 diagnoses) was estimated using the following formula, where RI was the observed relative incidence [40,41]:


$$attributable\;risk=\;\frac{AE\;cases\;in\;risk\;window}{number\;of\;eligible\;COVID-19\;cases\;in\;analysis}\times (1-\frac{1}{RI})\;$$

All analyses were conducted with SAS version 9.4 (SAS Institute Inc., Cary, NC, USA) and R version 4.1.2 and 4.1.3 (R Core Team 2021).

## Results

For the cohort analyses, we identified 319,300 eligible individuals in MarketScan and 1,017,410 in Medicare with a COVID-19 diagnosis during the study period and matched those patients to comparators without a COVID-19 diagnosis (S4 Table). For the SCRI analyses, 330,799 eligible individuals in MarketScan and 855,065 in Medicare were identified with an eligible COVID-19 diagnosis (S5 Table). Characteristics of the individuals included in both analyses are presented in Table 2.

In the Medicare cohort analysis, 6.3% of individuals with a COVID-19 diagnosis failed to match (29.0% of individuals with hospitalized COVID-19 failed to match), whereas in the MarketScan cohort analysis, 10.9% of individuals with a COVID-19 diagnosis failed to match (68.4% of individuals with hospitalized COVID-19 failed to match) (S6 Table). In the matched cohorts after sIPT weighting, the characteristics of the COVID-19 group and the comparator group were well balanced in all AE-specific data sets, with ASDs less than 0.05 for all measured characteristics (S7 Fig). AEs were relatively rare in both data sources, particularly for GBS, encephalitis/encephalomyelitis, and transverse myelitis (Table 3); small case counts resulted in very small AE-specific analysis sets. The cumulative incidence of all AEs was higher in the older Medicare population (Table 3).

**Table 3:** Association of a COVID-19 diagnosis with adverse events, cohort design, follow-up starting on the day after time 0.

Adverse event	Data source	Exposure group	Adverse event cases	Person-time (days)	Crude cumulative incidence^ a^ (95% CI)	Crude HR (95% CI)	sIPT-weighted HR (95% CI)
Guillain-Barré syndrome	MarketScan	COVID-19	10	26,740,943	5.12 (1.82-8.43)	9.99 (1.28-78.02)	9.57 (1.23-74.74)
		Comparator	1	26,735,597	1.48 (0.00-4.37)	—	—
	Medicare	COVID-19	31	80,000,492	5.06 (3.14-6.98)	1.93 (1.02-3.64)	1.97 (1.04-3.74)
		Comparator	17	86,246,732	13.59 (0.00-34.59)	—	—
Bell’s palsy	MarketScan	COVID-19	109	26,710,491	112.77 (72.11-153.41)	1.21 (0.91-1.61)	1.13 (0.85-1.50)
		Comparator	90	26,715,066	69.66 (50.80-88.51)	—	—
	Medicare	COVID-19	756	79,823,029	208.27 (187.29-229.25)	1.18 (1.05-1.31)	1.11 (1.00-1.24)
		Comparator	690	86,050,482	178.03 (156.21-199.85)	—	—
Encephalitis/ encephalomyelitis	MarketScan	COVID-19	11	26,738,915	5.28 (1.99-8.58)	2.20 (0.76-6.33)	1.99 (0.69-5.77)
		Comparator	5	26,737,424	6.17 (0.00-13.19)	—	—
	Medicare	COVID-19	109	80,018,307	27.43 (19.21-35.65)	1.43 (1.02-2.00)	1.36 (0.96-1.94)
		Comparator	81	86,269,166	16.45 (11.65-21.24)	—	—
Narcolepsy	MarketScan	COVID-19	32	26,713,507	39.46 (19.08-59.83)	1.33 (0.79-2.26)	1.21 (0.71-2.05)
		Comparator	24	26,712,918	25.32 (10.44-40.20)	—	—
	Medicare	COVID-19	169	79,956,203	45.05 (34.55-55.56)	1.21 (0.96-1.54)	1.11 (0.88-1.42)
		Comparator	149	86,209,713	49.80 (16.16-83.42)	—	—
Immune thrombocytopenia	MarketScan	COVID-19	42	26,717,156	27.58 (17.99-37.16)	2.10 (1.23-3.58)	2.06 (1.20-3.53)
		Comparator	20	26,720,031	16.54 (7.76-25.33)	—	—
	Medicare	COVID-19	497	79,815,901	108.20 (95.62-120.78)	1.40 (1.22-1.61)	1.36 (1.18-1.57)
		Comparator	377	86,057,511	103.37 (78.81-127.93)	—	—
Transverse myelitis	MarketScan	COVID-19	2	26,740,060	0.68 (0.00-1.62)	2.00 (0.18-22.04)	2.30 (0.21-25.41)
		Comparator	1	26,737,141	0.56 (0.00-1.64)	—	—
	Medicare	COVID-19	< 11	80,044,399	1.15 (0.13-2.18)	0.89 (0.27-2.94)	0.93 (0.28-3.09)
		Comparator	< 11	86,296,624	1.14 (0.14-2.14)	—	—

CI = confidence interval; COVID-19 = coronavirus disease 2019; HR = hazard ratio; sIPT = stabilized inverse probability of treatment.

— denotes the reference group.

^a^Estimated as the 1 minus the Kaplan-Meier survival estimator; expressed as risk per 100,000 individuals.

Note: Privacy rules for Medicare require masking cell sizes of fewer than 11 individuals.

**Fig 3:**
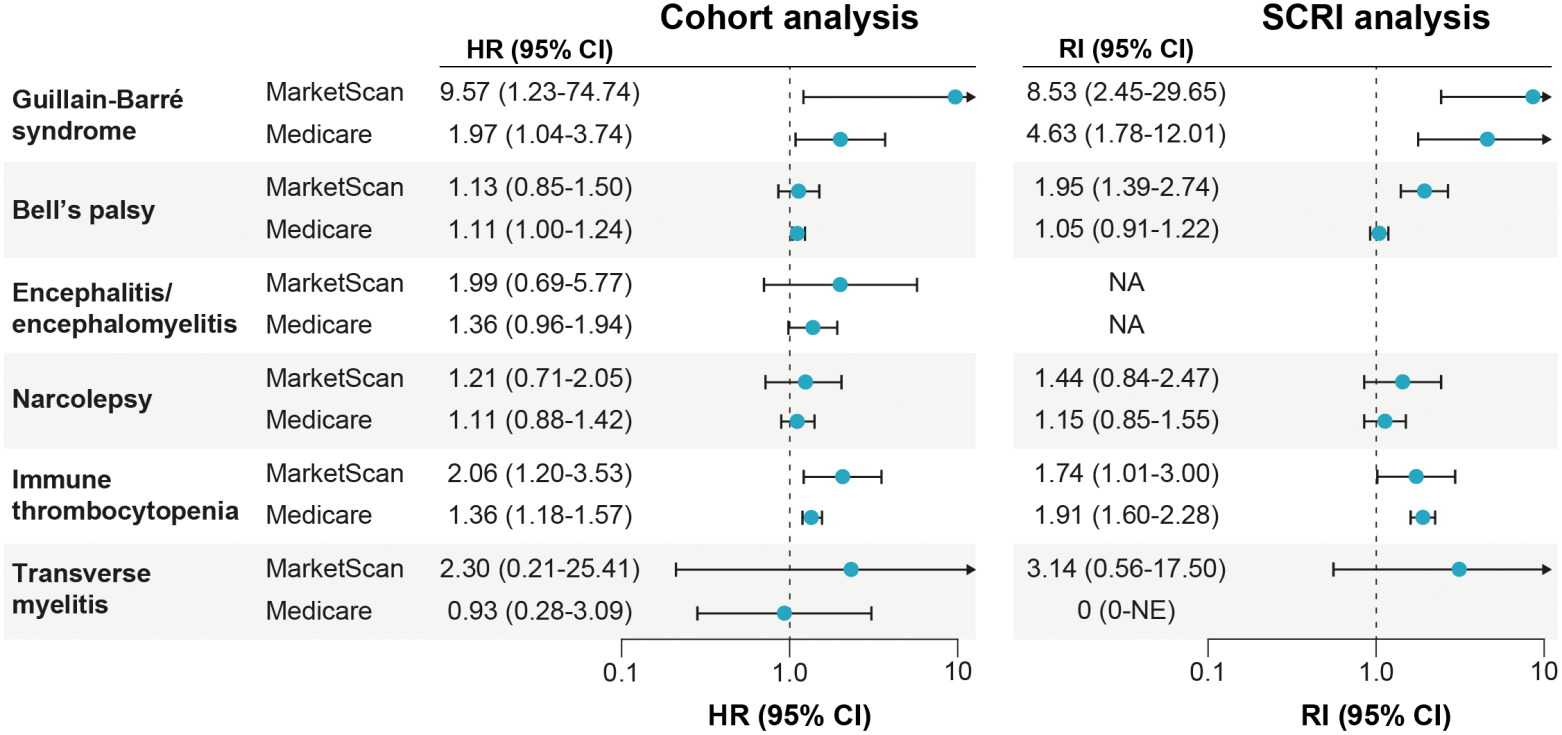
Estimated Association of a COVID-19 Diagnosis With Neurologic or Immune-Mediated Outcomes: Analysis Starting Follow-Up on the Day After Time 0. CI = confidence interval; HR = hazard ratio; NA = not applicable; NE = not estimable; RI = relative incidence; SCRI = self-controlled risk interval. Note: Encephalitis/encephalomyelitis was not evaluated with the SCRI design because of its known high case fatality rate.

Both study designs found increased HR and RI estimates for the association between COVID-19 diagnoses and GBS and ITP (summary in Fig 3; complete details given in Tables 3,4). The effect estimates for the association between COVID-19 diagnosis and GBS were largest in the younger MarketScan cohort with an HR of 9.57 (95% CI, 1.23–74.74), and an RI of 8.53 (95% CI, 2.45–29.65) using the SCRI approach. Although the effect sizes were smaller, estimates in the older Medicare population also displayed an association, with a cohort HR of 1.97 (95% CI, 1.04–3.74) and RI of 4.63 (95% CI, 1.78–12.01) for the SCRI analysis. Across populations and analytic approaches, a COVID-19 diagnosis also had a modest association with ITP, with more similar estimates across populations. In the cohort analysis, the HR for COVID-19 and ITP was 2.06 (95% CI, 1.20–3.53) in MarketScan and 1.36 (95% CI, 1.18–1.57) in Medicare. Similarly, the SCRI analysis yielded an RI of 1.74 (95% CI, 1.01–3.00) in MarketScan and 1.91 (95% CI, 1.60–2.28) in Medicare.

**Table 4:** Association of a COVID-19 diagnosis with adverse events, SCRI design, marketscan and medicare, follow-up starting on the day after time 0.

Adverse event	Data source	Total number of cases	Cases in risk window	Cases in reference window	RI (95% CI) ^a^
Guillain-Barré syndrome	MarketScan	10	5	5	8.53 (2.45-29.65)
	Medicare	35	17	18	4.63 (1.78-12.01)
Bell’s palsy	MarketScan	177	47	130	1.95 (1.39-2.74)
	Medicare	1,283	253	1,030	1.05 (0.91-1.22)
Narcolepsy	MarketScan	81	17	64	1.44 (0.84-2.47)
	Medicare	292	67	225	1.15 (0.85-1.55)
Immune thrombocytopenia	MarketScan	67	18	49	1.74 (1.01-3.00)
	Medicare	720	251	469	1.91 (1.60-2.28)
Transverse myelitis	MarketScan	6	2	4	3.14 (0.56-17.50)
	Medicare	13	0	13	0 (0-NE)

CI = confidence interval; COVID-19 = coronavirus disease 2019; NE = not estimable; RI = relative incidence; SCRI = self-controlled risk interval.

^a^Conditional Poisson model accounting for event-dependent observation windows and adjusted for seasonality.

Estimates for other outcomes were generally modest and varied by data source and study design. A diagnosis of COVID-19 showed some evidence of a potential association with Bell’s palsy in the MarketScan and Medicare cohort analyses (HRs of 1.13 [95% CI, 0.85–1.50] and 1.11 [95% CI, 1.00–1.24], respectively) and in the MarketScan and Medicare SCRI analyses as well (RIs of 1.95 [95% CI, 1.39–2.74] and 1.05 [95% CI, 0.91–1.22]). Encephalitis/encephalomyelitis demonstrated potential associations with COVID-19 in both cohort analyses.

The SCRI analyses were used to estimate attributable risks, estimating the absolute number of excess cases of the AEs resulting from COVID-19 diagnoses. Because most of the AEs were very rare, attributable risks were generally quite small. The largest observed attributable risk was 13.98 ITP cases per 100,000 COVID-19 diagnoses in the older Medicare population (S8 Table).

Estimates from the additional analyses starting follow-up/risk windows on Time 0 (S9-S10 Tables) largely followed the trends of the estimates from the analyses starting follow-up the day after Time 0. While similar in trend, the effect measure estimates starting follow-up on Time 0 were usually larger compared to those starting follow-up on the day after Time 0, with the exception of the Medicare cohort analyses for GBS and Bell’s palsy in which the Time 0 estimates were attenuated. Only the Medicare SCRI estimates for transverse myelitis varied substantially between analyses; the analyses starting the risk window on Time 0 resulted in an RI estimate of 3.76 (95% CI, 1.25–11.35), but the analyses starting the risk window on the day after Time 0 resulted in an RI estimate of 0 (95% CI, 0-not estimable) because there were no cases in the risk window.

## Discussion

In our analyses of large cohorts of adult patients across age groups and data sources, estimates from all data sources and study designs demonstrated reasonably strong associations between COVID-19 diagnosis and the incidence of GBS, and more modest associations between COVID-19 diagnosis and ITP. Evidence for an association of a COVID-19 diagnosis with the remaining AEs (Bell’s palsy, encephalitis/encephalomyelitis, narcolepsy, and transverse myelitis) was more mixed, however; the observed effect measure estimates were generally above the null, although they were modest, imprecise, or varied by data source and study design.

The observed association was strongest between GBS and COVID-19 diagnosis, with the highest effect measure estimates identified in the MarketScan analyses. The older Medicare population had higher incidence of GBS and most other AEs compared with the younger MarketScan population [23]. Thus, even modest relative increases in risk in the older Medicare population may correspond to large absolute attributable risk estimates (e.g., despite much larger RI estimates in MarketScan compared with Medicare [8.53 vs. 4.63], the attributable risk estimates were similar across both data sources [1.33 vs. 1.56 cases per 100,000 COVID-19 cases]). The observed association between a COVID-19 diagnosis and GBS is consistent with prior studies that identified consistent trends regarding the development of GBS following a recent SARS-CoV-2 infection [3,5,6,42–44]. However, few systematic reviews have been able to estimate the strength of the association between GBS and COVID-19 because of the heterogenous definitions of GBS and COVID-19, challenges identifying asymptomatic infections, lack of uniformity in case report data, and inability to establish the temporality of events [3,5,42–44].

Although not as strong as the observed positive association between COVID-19 diagnosis and GBS, this analysis identified a consistent, modest relationship between COVID-19 diagnosis and ITP across study designs and data sources. Because of the relatively high incidence of ITP in the older Medicare population, the modest RI observed in Medicare corresponded to the largest attributable risk estimate observed for these outcomes. In existing literature, elderly and severely ill patients with COVID-19 display consistently increased risks of ITP [13,45,46]. However, little evidence exists regarding the causal relationship between COVID-19 diagnosis and ITP, outside of the association between platelet levels and COVID-19 disease severity and an observed latency period (18–21 days) between COVID-19 diagnosis and the development of ITP [47].

Outside of GBS and ITP, the observed estimates in these analyses were generally consistent with a potential relationship between COVID-19 diagnosis and Bell’s palsy, although estimates were somewhat inconsistent across data sources and study designs. The largest effect size estimate was for the SCRI design in MarketScan (RI = 1.95, 95% CI, 1.39–2.74), while the SCRI estimate in Medicare was generally null; the cohort HR estimates were both generally modest. However, a potential relationship with Bell’s palsy identified within this analysis is consistent with findings of other research [48,49]. Given the longer time period for neurologic conditions to manifest, the findings within this report potentially underestimated this association given the analyses’ short follow-up period [6,48,50,51]. Cohort HR estimates for encephalitis/encephalomyelitis were elevated although imprecise (particularly in the younger MarketScan population with a small number of cases). Previous reviews have identified that older individuals and those with more severe COVID-19 cases were at greater risk of developing encephalitis following COVID-19 infection [10,52,53]. Estimates for narcolepsy from both study designs and data sources were all modestly above the null. And for transverse myelitis, small case counts resulted in inconsistent and imprecise estimates; however, existing case reports and systematic analyses observed development of transverse myelitis after COVID-19 diagnosis, suggesting a potential association between COVID-19 and subsequent onset of transverse myelitis [9,54,55].

The strengths of this study include study designs and analytical methods designed to mitigate confounding, exposure misclassification, selection bias, and reverse causality; a large and diverse US study population; a requirement that individuals in the cohort analysis have at least one claim in the 12 months prior to Time 0 to balance healthcare-seeking behavior across groups; and examination of several of immunologic and neurologic outcomes. The large sample sizes allowed for the precise estimations of the associations between COVID-19 diagnosis and several rare AEs given the large, diverse populations available in the MarketScan and Medicare data sources. We employed two distinct study designs with complementary properties; our cohort approach used exact matching and propensity score weighting to compare individuals diagnosed with COVID-19 to similar individuals who had not been diagnosed with COVID-19. Additionally, the cohort design matched the COVID-19 and comparator groups on exact calendar date, reducing the impact of calendar time–related confounding (e.g., seasonal trends or changes in healthcare utilization). The SCRI design is less vulnerable to bias from misclassification of exposure status because it only considers patients diagnosed with COVID-19; also, comparisons were made within an individual and were less vulnerable to confounding by patient characteristics. We also adjusted for potential time-varying confounding by controlling for seasonality. However, the 2 study designs do address the study question with different approaches and estimate different parameters; whereas the direction of effect measure estimates was consistent across analyses, the magnitude and precision of the effect measure estimates frequently differed across study designs. The SCRI was restricted to a fixed, prespecified risk interval, but the cohort approach used all available follow-up time in the study period, identifying more cases in the longer follow-up period than the SCRI. By leveraging multiple data sources and study designs, it is possible to compare and identify trends across multiple effect estimates and populations for each outcome.

Our study has several limitations, including its use of only insured populations (commercial insurance for those ages <65 years or Medicare fee-for-service for those aged ≥65 years) that may be healthier and more likely to seek care than uninsured persons. We use ICD-10-CM diagnosis codes to define outcomes, and misclassification of outcomes is possible. A prior study from the BEST Initiative found low positive predictive values for Bell’s palsy (12.66%; 95% CI, 7.02%−21.76%) and ITP (4.00%; 95% CI, 1.37%−11.11%) in Medicare following COVID-19 vaccination [56]. We did not adjudicate outcome diagnoses using medical record review because medical charts were not available; therefore, we do not know if all identified outcomes are true outcomes. Diagnosis dates in the claims data represent the date the disease was identified by a healthcare professional, and it may not correspond with the onset of illness. In the SCRI analyses, our washout and risk widows may be conservative; our washout window may extend into a period in which COVID-19-mediated AEs are possible, and our risk windows may not extend far enough to capture all COVID-19-mediated events. A risk window of 41 days was used for all outcomes, similar to many COVID-19 vaccine surveillance activities performed at the time of the analyses which used 41−42 days; however, the biologically relevant risk period for COVID-19 illness (where the date of onset of disease and duration of illness may not align with the recorded diagnosis date) may differ from the relevant period following vaccination. The cohort results may not be generalizable to all COVID-19 cases during the study period because a disproportionately large share of the COVID-19 group hospitalized at Time 0 was excluded because of failure to match to a comparator; thus, these results may overrepresent nonhospitalized cases. The cohort design may be subject to bias from confounding between groups resulting from differences in characteristics, including risk factors for COVID-19 and AEs, healthcare-seeking behavior, or access to healthcare. In this study, only relatively small differences between exposure groups were noted for clinical characteristics. Confounding by measured covariates was addressed through matching and weighting approaches, and all characteristics were well-balanced after weighting, but the potential for confounding by unmeasured characteristics remained.

For both the cohort and SCRI designs, because of potential selection bias and reverse causality involving cases occurring on Time 0, we focused on the analyses starting the risk windows and follow-up on the day after the COVID-19 diagnosis or matched comparator date, resulting in the exclusion of a large number of individuals with AEs that occurred on Time 0 [38]. In the cohort analysis, hospitalization status on Time 0 was one of the matching criteria, so hospitalized COVID-19 patients were matched to hospitalized comparators on the date of admission or evidence of any inpatient claims on Time 0; thus, starting follow-up on the day after Time 0 avoided selection bias because of conditioning on hospitalization, where hospital-based comparators may have been more likely to be hospitalized for serious events, potentially inflating the AE risk in the comparator group. Additionally, in both the SCRI and cohort analyses, although there were likely true COVID-19-associated AEs occurring on the same day as their COVID-19 diagnosis, there were also AE cases on Time 0 where a COVID-19 diagnosis was likely incidental or secondary to seeking care for the AE (i.e., a form of reverse causality) [38], artificially inflating the number of AE cases identified during the SCRI risk window or in the cohort COVID-19-diagnosed group. These phenomena may affect outcome events, study designs, and data sources differently, particularly during a time of pandemic-induced reductions in healthcare utilization. Because of these issues, we chose to prioritize methods that reduced the likelihood of selection bias and reverse causality, at the expense of complete case ascertainment [38]. The consequence of this decision is a loss of precision and potential lack of generalizability of the study results because a large number of AE cases (i.e., those occurring on Time 0) were excluded. However, while the estimates from analyses starting follow-up on the day after Time 0 were somewhat attenuated relative to those starting on Time 0, the results of both sets of analyses were generally consistent. Although the initial study protocol [22] designated the primary analyses as those starting follow-up on Time 0 and the secondary analyses as those starting follow-up the day after Time 0, given the biases described here, the analyses starting follow-up on the day after Time 0 are considered the more reliable and clinically relevant results.

## Conclusions

These findings contribute to a growing body of research detailing the complex, adverse health effects of COVID-19 in both the short and long term. In this analysis, modest to strong associations were found between COVID-19 diagnosis and GBS and ITP. Although the presence of associations between COVID-19 and other neurologic or immune-mediated AEs cannot be ruled out, no consistent relationships were found in these analyses. Further analysis is needed to understand the health impact of COVID-19 diagnosis in the context of the current variants and vaccine environment, and with a longer follow-up period to capture longer term impacts.

## Supporting information

S1 Table Code Lists for Select Key Variables. (DOCX)

S2 Table Operational Definitions for Identification of Outcomes in Healthcare Claims Data. (DOCX)

S3 Table Covariates for AE-Specific Propensity Score Models. (DOCX)

S4 Table Selection of Individuals With a COVID-19 Diagnosis and Comparator Individuals Without a COVID-19 Diagnosis for the Cohort Study. (DOCX)

S5 Table Selection of Individuals With a COVID-19 Diagnosis for the Self-Controlled Risk Interval Study. (DOCX)

S6 Table Characteristics of Individuals With a COVID-19 Diagnosis Included in the Cohort Study and Those Excluded for Failing to Match. (DOCX)

S7 Fig Balance of Covariate Distributions Among Individuals With a COVID-19 Diagnosis and Comparator Individuals Without a COVID-19 Diagnosis, Cohort Design, Before and After Stabilized Inverse Probability of Treatment Weighting. (DOCX)

S8 Table Estimated Attributable Risk of Adverse Events Associated With COVID-19 Diagnoses. (DOCX)

S9 Table Association of a COVID-19 Diagnosis With Adverse Events, Cohort Design, Follow-Up Starting on Time 0. (DOCX)

S10 Table Association of a COVID-19 Diagnosis With Adverse Events, SCRI Design, MarketScan and Medicare, Follow-Up Starting on Time 0. (DOCX)

S1 File STROBE-checklist. (DOC)
