# Supplementary material for: Risk of neurologic or immune-mediated adverse events after COVID-19 diagnosis in the United States
Source: PLoS One. 2025 Nov 24;20(11):e0333704. doi: 10.1371/journal.pone.0333704 (PMC12643290; doi:10.1371/journal.pone.0333704)
Supplement: S1 Table — (DOCX) [file pone.0333704.s001.docx]

S1 Table. Code Lists for Select Key Variables

| Variable | Code type | Code | Code description |
| --- | --- | --- | --- |
| COVID-19 | ICD-10-CM | U07.1 | COVID-19 |
| History of COVID-19 or select respiratory infection | ICD-10-CM | B97.21 | SARS-associated coronavirus as the cause of diseases classified elsewhere |
|  | ICD-10-CM | B97.29 | Other coronavirus as the cause of diseases classified elsewhere |
|  | ICD-10-CM | J12.81 | Pneumonia due to SARS-associated coronavirus |
|  | ICD-10-CM | U07.1 | COVID-19 |
| Guillain-Barré Syndrome | ICD-10-CM | G61.0 | Guillain-Barré syndrome |
| Bell’s palsy | ICD-10-CM | G51.0 | Bell's palsy |
|  | ICD-10-CM | G51.8 | Other disorders of facial nerve |
|  | ICD-10-CM | G51.9 | Disorder of facial nerve, unspecified |
| Encephalitis/ encephalomyelitis | ICD-10-CM | G04.00 | Acute disseminated encephalitis and encephalomyelitis, unspecified |
|  | ICD-10-CM | G04.02 | Postimmunization acute disseminated encephalitis, myelitis and encephalomyelitis |
|  | ICD-10-CM | G04.81 | Other encephalitis and encephalomyelitis |
|  | ICD-10-CM | G04.90 | Encephalitis and encephalomyelitis, unspecified |
|  | ICD-10-CM | G05.3 | Encephalitis and encephalomyelitis in diseases classified elsewhere |
| Narcolepsy | ICD-10-CM | G47.411 | Narcolepsy with cataplexy |
|  | ICD-10-CM | G47.419 | Narcolepsy without cataplexy |
|  | ICD-10-CM | G47.421 | Narcolepsy in conditions classified elsewhere with cataplexy |
|  | ICD-10-CM | G47.429 | Narcolepsy in conditions classified elsewhere without cataplexy |
| Immune thrombocytopenia | ICD-10-CM | D69.3 | Immune thrombocytopenic purpura |
| Transverse myelitis | ICD-10-CM | G37.3 | Acute transverse myelitis in demyelinating disease of central nervous system |

COVID-19 = coronavirus disease 2019; ICD-10-CM = *International Classification of Diseases, 10^th^ Revision, Clinical Modification*; SARS = severe acute respiratory syndrome.
