# Supplementary material for: Risk of neurologic or immune-mediated adverse events after COVID-19 diagnosis in the United States
Source: PLoS One. 2025 Nov 24;20(11):e0333704. doi: 10.1371/journal.pone.0333704 (PMC12643290; doi:10.1371/journal.pone.0333704)
Supplement: S2 Table — (DOCX) [file pone.0333704.s002.docx]

S2 Table. Operational Definitions for Identification of Outcomes in Healthcare Claims Data

| Outcome name | Type of outcome | Washout window, days^a^ | Care setting | Code type | Diagnosis position | Prespecified |
| --- | --- | --- | --- | --- | --- | --- |
| Guillain-Barré syndrome | Time-to-event^b^, binary^c^ | [−365 to −1] in IP, OP/PB settings | IP | ICD-10-CM | Principal | Yes |
| Bell’s palsy | Time-to-event^b^, binary^c^ | [−183 to −1] | IP, OP/PB | ICD-10-CM | Any^d^ | Yes |
| Encephalitis/encephalomyelitis | Time-to-event^b^, binary^c^ | [−183 to −1] in IP, OP/PB settings | IP | ICD-10-CM | Any^d^ | Yes |
| Narcolepsy | Time-to-event^b^, binary^c^ | [−365 to −1] | IP, OP/PB | ICD-10-CM | Any^d^ | Yes |
| Immune thrombocytopenia | Time-to-event^b^, binary^c^ | [−365 to −1] | IP, OP/PB | ICD-10-CM | Any^d^ | Yes |
| Transverse myelitis | Time-to-event^b^, binary^c^ | [−365 to −1] in IP, OP/PB settings | IP, OP-ED | ICD-10-CM | Any^d^ | Yes |

AE = adverse event; ED = emergency department; ICD‑10‑CM = *International Classification of Diseases, 10th Revision, Clinical Modification*; IP = inpatient; OP = outpatient; PB = professional/provider; SCRI = self-controlled risk interval.

^a^ For the cohort analysis, the washout window is applied before the index date (Time 0). For the SCRI analysis, the washout window is applied before the AE event date.

^b^ For the cohort analysis.

^C^ For the SCRI.

^d^ Any diagnosis position, excluding admitting diagnosis (available in Medicare only).

Note: Complete details of operational definitions for all study variables are given in the publicly available study protocol: CBER Surveillance Program. Evaluating the Risk of Adverse Events After COVID-19 Diagnosis: protocol. U.S. Food & Drug Administration; 10 February 2023. Available at: <https://bestinitiative.org/wp-content/uploads/2023/02/BEST-Post-COVID-19-AE-Protocol_2023.pdf>. Accessed 2 March 2023.
