# Supplementary material for: Risk of neurologic or immune-mediated adverse events after COVID-19 diagnosis in the United States
Source: PLoS One. 2025 Nov 24;20(11):e0333704. doi: 10.1371/journal.pone.0333704 (PMC12643290; doi:10.1371/journal.pone.0333704)
Supplement: S3 Table — (DOCX) [file pone.0333704.s003.docx]

S3 Table. Covariates for AE-Specific Propensity Score Models

| Covariate | Model form | Guillain-Barré syndrome | Bell's palsy | ENC | Narcolepsy | ITP | TM |
| --- | --- | --- | --- | --- | --- | --- | --- |
| Age^a^ | Age, age^2^, age^3^ | X | X | X | X | X | X |
| Sex^a^ | Binary | X | X | X | X | X | X |
| County/MSA and state of residence^a^ | Categorical, indicator for each level | X | X | X | X | X | X |
| Race/ethnicity^c^ | Categorical, indicator for each level | X | X | X | X | X | X |
| Dual Medicare/Medicaid eligibility^b^ | Binary | X | X | X | X | X | X |
| Reason for entering Medicare^b^ | Categorical, indicator for each level | X | X | X | X | X | X |
| Hospitalization status on Time 0^a^ | Binary | X | X | X | X | X | X |
| SNF/LTC residence on Time 0^a, b^ | Binary | X | X | X | X | X | X |
| Animal exposure/bites or rabies | Binary |  |  | X |  | X |  |
| Antiphospholipid syndrome | Binary |  |  |  |  | X | X |
| Autoimmune disorders | Binary | X | X | X |  | X | X |
| Brain lesions related to secondary narcolepsy | Binary |  |  |  | X |  |  |
| Cancer | Binary |  | X |  |  | X |  |
| Chronic lymphocytic leukemia | Binary |  |  |  |  | X |  |
| Chronic lung disease | Binary |  |  |  |  |  | X |
| Dementia/neurological conditions | Binary | X | X | X | X |  | X |
| Diabetes mellitus, type 1 or 2 | Binary |  | X |  |  |  | X |
| Hemiplegia/paraplegia | Binary | X |  |  |  |  | X |
| Herpes simplex virus infection | Binary | X | X | X |  | X |  |
| Immunocompromised state^a^ | Binary | X | X | X | X | X | X |
| Infection associated with GBS | Binary | X |  |  |  |  |  |
| Infection associated with ITP | Binary |  |  |  |  | X |  |
| Inpatient surgery | Binary | X |  |  |  |  |  |
| Mental health conditions | Binary |  | X |  | X |  |  |
| Nutritional deficiencies | Binary |  |  |  |  |  | X |
| Obesity | Binary | X | X |  | X |  |  |
| Pneumonia or lower respiratory tract infection | Binary | X |  | X |  | X | X |
| Pregnancy^c^ | Binary |  | X |  |  |  |  |
| Sepsis | Binary |  |  |  |  | X |  |
| Stroke/cerebrovascular disease | Binary |  |  |  | X |  |  |
| Trauma | Binary | X |  |  |  |  |  |
| Tuberculosis | Binary |  |  |  |  | X |  |
| Inpatient hospital stays before Time 0 | Categorical, indicator for each level | X | X | X | X | X | X |
| ED visits | Categorical, indicator for each level | X | X | X | X | X | X |
| Outpatient visits | Categorical, indicator for each level | X | X | X | X | X | X |
| SNF/LTC stay^d^ | Binary | X |  | X |  | X | X |
| Influenza vaccination in previous year | Binary | X | X | X | X | X | X |

AE = adverse event; ED = emergency department; ENC = encephalitis/encephalomyelitis; GBS = Guillain-Barré syndrome; HS = hemorrhagic stroke; ITP = immune thrombocytopenia; LTC = long-term care; MSA = metropolitan statistical area; SNF = skilled nursing facility; TM = transverse myelitis; TTS = thrombosis with thrombocytopenia syndrome.

^a^ Matching variables were included in all propensity score models.

^b^ Only included in Medicare.

^c^ Only included in MarketScan.

^d^ SNF and LTC both included in Medicare; only SNF included in MarketScan.

Sources: National Institute of Neurological Disorders and Stroke [1-5]; National Heart, Lung, and Blood Institute [6].

References:

1. NINDS. National Institutes of Health. Guillain-Barré syndrome. 31 July 2023. https://www.ninds.nih.gov/health-information/disorders/guillain-barre-syndrome. Accessed 2 November 2023.

2. NINDS. National Institutes of Health. Bell's palsy fact sheet. June 2018. https://catalog.ninds.nih.gov/sites/default/files/publications/bells-palsy.pdf. Accessed 9 February 2023.

3. NINDS. National Institutes of Health. Meningitis and encephalitis fact sheet. June 2018. https://catalog.ninds.nih.gov/sites/default/files/publications/meningitis-encephalitis.pdf. Accessed 9 February 2023.

4. NINDS. National Institutes of Health. Narcolepsy fact sheet. 25 April 2022. https://catalog.ninds.nih.gov/sites/default/files/publications/narcolepsy.pdf. Accessed 9 February 2023.

5. NINDS. National Institutes of Health. Transverse myelitis. 20 January 2023. https://www.ninds.nih.gov/health-information/disorders/transverse-myelitis. Accessed 2 November 2023.

6. NHLBI. National Institutes of Health. Immune thrombocytopenia (ITP). 2022. https://www.nhlbi.nih.gov/health/immune-thrombocytopenia#What-causes-ITP? Accessed 14 July 2022.
