# Supplementary material for: Risk of neurologic or immune-mediated adverse events after COVID-19 diagnosis in the United States
Source: PLoS One. 2025 Nov 24;20(11):e0333704. doi: 10.1371/journal.pone.0333704 (PMC12643290; doi:10.1371/journal.pone.0333704)
Supplement: S4 Table — (DOCX) [file pone.0333704.s004.docx]

S4 Table. Selection of Individuals With a COVID-19 Diagnosis and Comparator Individuals Without a COVID-19 Diagnosis for the Cohort Study

| Characteristic | Value | |
| --- | --- | --- |
|  | MarketScan | Medicare |
| Overall study cohort |  |  |
| Individuals with COVID-19 diagnosis during the study period, N | 569,754 | 1,807,670 |
| *Excluded for being aged outside age range, N (%)* | 53,723 (9.43%) | 253,377 (14.0%) |
| *Excluded for lacking 365 days of continuous database enrollment before Time 0, N (%)* | 142,532 (25.02%) | 436,595 (24.2%) |
| *Excluded for lacking ≥ 1 healthcare claim during 365 days before Time 0, N (%)* | 51,066 (8.96%) | 9,722 (0.5%) |
| *Excluded for having a COVID-19 diagnosis before Time 0, N (%)* | 7,002 (1.23%) | 10,510 (0.6%) |
| *Excluded for having a select respiratory infection before Time 0, N (%)* | 382 (0.07%) | 12,048 (0.7%) |
| Eligible adults with new-onset COVID-19, N | 358,306 | 1,085,418 |
| *Excluded for failing to match, N (%)* | 39,006 (10.89%) | 68,008 (6.3%) |
| Matched individuals with COVID-19, N | 319,300 | 1,017,410 |
| Matched comparator observations^a^ (unique individuals) | 319,300 (312,282) | 1,017,410 (930,513) |
| Total study cohort, N | 638,600 | 2,034,820 |
| Adverse event-specific analysis sets^b^ |  |  |
| *Excluded for having previous Guillain-Barré syndrome, N (%)* | 97 (0.02%) | 1,602 (0.1%) |
| Guillain-Barré syndrome analysis set, N | 638,503 | 2,033,218 |
| *Excluded for having previous Bell’s palsy, N (%)* | 501 (0.08%) | 4,560 (0.2%) |
| Bell’s palsy analysis set, N | 638,099 | 2,030,260 |
| *Excluded for having previous encephalitis/encephalomyelitis, N (%)* | 72 (0.01%) | 1,039 (0.1%) |
| Encephalitis/encephalomyelitis analysis set, N | 638,528 | 2,033,781 |
| *Excluded for having previous narcolepsy, N (%)* | 698 (0.11%) | 2,504 (0.1%) |
| Narcolepsy analysis set, N | 637,902 | 2,032,316 |
| *Excluded for having previous immune thrombocytopenia, N (%)* | 467 (0.07%) | 5,517 (0.3%) |
| Immune thrombocytopenia analysis set, N | 638,133 | 2,029,303 |
| *Excluded for having previous transverse myelitis, N (%)* | 106 (0.02%) | 532 (0.0%) |
| Transverse myelitis analysis set, N | 638,494 | 2,034,288 |

COVID-19 = coronavirus disease 2019.

^a^ A unique individual may have been included multiple times because of matching with replacement, and an individual may have been included in both the exposure and comparator groups. This table counts each instance of an individual’s entry separately as distinct observations; the number of unique individuals is reported separately.

^b^ Denominators for all adverse event-specific exclusions are the total study cohort; exclusions are noncumulative across analysis sets.
