# Supplementary material for: Risk of neurologic or immune-mediated adverse events after COVID-19 diagnosis in the United States
Source: PLoS One. 2025 Nov 24;20(11):e0333704. doi: 10.1371/journal.pone.0333704 (PMC12643290; doi:10.1371/journal.pone.0333704)
Supplement: S5 Table — (DOCX) [file pone.0333704.s005.docx]

S5 Table. Selection of Individuals With a COVID-19 Diagnosis for the Self-Controlled Risk Interval Study

| Characteristic | Value | |
| --- | --- | --- |
|  | MarketScan | Medicare |
| Overall SCRI population |  |  |
| Individuals with COVID-19 diagnosis during the study period | 509,875 | 1,571,901 |
| *Excluded for being aged outside age range, N (%)* | 51,848 (10.2%) | 219,427 (14.0%) |
| *Excluded for lacking 365 days of continuous database enrollment before Time 0, N (%)* | 128,310 (25.2%) | 366,363 (23.3%) |
| *Excluded for having a previous COVID-19 diagnosis, N (%)* | 2,135 (0.4%) | 85,728 (5.5%) |
| *Excluded for having a previous select respiratory infection, N (%)* | 119 (0.0%) | 407 (0.0%) |
| *Excluded for lacking ≥ 1 day in the pre-exposure reference window, N (%)* | 25,306 (5.0%) | 44,911 (2.9%) |
| Total SCRI population, N | 330,799 | 855,065 |
| Adverse event-specific analysis sets^a^ |  |  |
| *Excluded for not having Guillain-Barré syndrome in risk or reference window, N (%)* | 330,779 (100.0%) | 854,999 (100.0%) |
| *Excluded for lacking 365 days of continuous database enrollment before outcome date, N (%)* | 0 (0.0%) | 0 (0.0%) |
| *Excluded for having Guillain-Barré syndrome in outcome washout window, N (%)* | 5 (0.0%) | 19 (0.0%) |
| Guillain-Barré syndrome analysis set, N | 12 | 47 |
| *Excluded for not having Bell’s palsy in risk or reference window, N (%)* | 330,507 (99.9%) | 853,077 (99.8%) |
| *Excluded for lacking 183 days of continuous database enrollment before outcome date, N (%)* | 0 (0.0%) | < 11 |
| *Excluded for having Bell’s palsy in outcome washout window, N (%)* | 61 (0.0%) | > 11 |
| Bell’s palsy analysis set, N | 192 | 1,480 |
| *Excluded for not having narcolepsy in risk or reference window, N (%)* | 330,506 (99.9%) | 854,206 (99.9%) |
| *Excluded for lacking 365 days of continuous database enrollment before outcome date, N (%)* | 0 (0.0%) | < 11 |
| *Excluded for having narcolepsy in outcome washout window, N (%)* | 197 (0.1%) | > 11 |
| Narcolepsy analysis set, N | 86 | 338 |
| *Excluded for not having immune thrombocytopenia in risk or reference window, N (%)* | 330,599 (99.9%) | 853,053 (99.8%) |
| *Excluded for lacking 365 days of continuous database enrollment before outcome date, N (%)* | 0 (0.0%) | 0 (0.0%) |
| *Excluded for having immune thrombocytopenia in outcome washout window, N (%)* | 111 (0.0%) | 1,092 (0.1%) |
| Immune thrombocytopenia analysis set, N | 78 | 920 |
| *Excluded for not having transverse myelitis in risk or reference window, N (%)* | 330,785 (100.0%) | 854,995 (100.0%) |
| *Excluded for lacking 365 days of continuous database enrollment before outcome date, N (%)* | 0 (0.0%) | 0 (0.0%) |
| *Excluded for having transverse myelitis in outcome washout window, N (%)* | 5 (0.0%) | 48 (0.0%) |
| Transverse myelitis analysis set, N | 7 | 22 |

COVID-19 = coronavirus disease 2019; SCRI = self-controlled risk interval.

^a^ Denominators for all outcome-specific exclusions are the total SCRI after excluding those with ≥ 1 day in risk and reference windows; exclusions are noncumulative across analysis sets.

Note: Privacy rules for Medicare require masking cell sizes containing fewer than 11 individuals.
