# Supplementary material for: Risk of neurologic or immune-mediated adverse events after COVID-19 diagnosis in the United States
Source: PLoS One. 2025 Nov 24;20(11):e0333704. doi: 10.1371/journal.pone.0333704 (PMC12643290; doi:10.1371/journal.pone.0333704)
Supplement: S6 Table — (DOCX) [file pone.0333704.s006.docx]

S6 Table. Characteristics of Individuals With a COVID-19 Diagnosis Included in the Cohort Study and Those Excluded for Failing to Match

A. MarketScan

| Characteristic | COVID-19 diagnosis (exposure) group retained by matching | Individuals with a COVID-19 diagnosis who failed to match |
| --- | --- | --- |
| Total unique individuals | 319,300 | 39,006 |
| Demographic characteristics |  |  |
| Age, years |  |  |
| Mean (SD) | 41.8 (13.6) | 44.1 (13.6) |
| Median (Q1, Q3) | 43 (30, 53) | 47 (33, 56) |
| Age groups, N (%) |  |  |
| 18-34 years | 105,842 (33.15) | 10,485 (26.88) |
| 35-49 years | 101,071 (31.65) | 12,060 (30.92) |
| 50-64 years | 112,387 (35.20) | 16,461 (42.20) |
| Sex, N (%) |  |  |
| Female | 178,975 (56.05) | 22,793 (58.43) |
| Male | 140,325 (43.95) | 16,213 (41.57) |
| US geographic region, N (%) |  |  |
| Northeast | 62,215 (19.48) | 949 (2.43) |
| North central | 71,170 (22.29) | 1,788 (4.58) |
| South | 149,868 (46.94) | 26,883 (68.92) |
| West | 36,037 (11.29) | 9,057 (23.22) |
| Unknown | 10 (0.00) | 329 (0.84) |
| Hospitalized on Time 0, N (%) | 4,046 (1.27) | 8,738 (22.40) |
| Comorbidities, N (%) |  |  |
| Animal exposure/bites or rabies | 976 (0.31) | 124 (0.32) |
| Antiphospholipid syndrome | 160 (0.05) | 28 (0.07) |
| Autoimmune disorders | 12,677 (3.97) | 1,759 (4.51) |
| Brain lesions related to secondary narcolepsy | 30 (0.01) | 2 (0.01) |
| Cancer | 13,421 (4.20) | 1,987 (5.09) |
| Chronic lymphocytic leukemia | 213 (0.07) | 41 (0.11) |
| Chronic kidney disease or renal disease (other than end-stage renal disease) | 6,052 (1.90) | 1,495 (3.83) |
| Chronic liver disease | 10,763 (3.37) | 1,566 (4.01) |
| Chronic lung diseases | 32,616 (10.21) | 4,285 (10.99) |
| Dementia or other neurologic condition | 19,722 (6.18) | 2,551 (6.54) |
| Diabetes, Type 1 or 2 | 33,117 (10.37) | 6,374 (16.34) |
| Disseminated intravascular coagulation^a^ | 24 (0.01) | 9 (0.02) |
| Heart conditions | 37,470 (11.74) | 5,494 (14.09) |
| Hemiplegia or paraplegia | 857 (0.27) | 202 (0.52) |
| Herpes simplex infection | 6,082 (1.90) | 709 (1.82) |
| Hypertension | 78,717 (24.65) | 13,371 (34.28) |
| Immunocompromised state | 37,672 (11.80) | 5,422 (13.90) |
| Infection associated with GBS | 17,961 (5.63) | 2,091 (5.36) |
| Viral infection associated with ITP | 1,290 (0.40) | 209 (0.54) |
| Infection associated with myocarditis/pericarditis | 16,753 (5.25) | 2,322 (5.95) |
| Inpatient surgery | 1,985 (0.62) | 379 (0.97) |
| Lipid abnormality | 73,480 (23.01) | 10,908 (27.96) |
| Mental health conditions | 73,913 (23.15) | 10,422 (26.72) |
| Nutritional deficiency | 2,088 (0.65) | 374 (0.96) |
| Obese or severely obese | 63,585 (19.91) | 9,775 (25.06) |
| Peripheral vascular disease | 4,393 (1.38) | 733 (1.88) |
| Pneumonia or lower respiratory tract infection | 41,355 (12.95) | 5,598 (14.35) |
| Pregnancy | 2,616 (0.82) | 266 (0.68) |
| Sepsis | 1,277 (0.40) | 340 (0.87) |
| Sickle cell disease or thalassemia | 750 (0.23) | 112 (0.29) |
| Smoking/nicotine dependency | 11,536 (3.61) | 1,391 (3.57) |
| Stroke or cerebrovascular disease | 4,730 (1.48) | 715 (1.83) |
| Thromboembolism | 3,531 (1.11) | 642 (1.65) |
| Thrombophilia | 1,096 (0.34) | 137 (0.35) |
| Trauma | 204 (0.06) | 38 (0.10) |
| Tuberculosis | 138 (0.04) | 21 (0.05) |
| Healthcare utilization, N (%) |  |  |
| Inpatient stays in previous 365 days |  |  |
| 0 | 305,018 (95.53) | 36,528 (93.65) |
| 1-2 | 13,162 (4.12) | 2,138 (5.48) |
| ≥ 3 | 1,120 (0.35) | 340 (0.87) |
| ED visits in previous 365 days |  |  |
| 0 | 255,758 (80.10) | 30,760 (78.86) |
| 1-2 | 54,798 (17.16) | 7,022 (18.00) |
| ≥ 3 | 8,744 (2.74) | 1,224 (3.14) |
| Outpatient provider visits^b^ in previous 365 days |  |  |
| 0 | 2,076 (0.65) | 248 (0.64) |
| 1-2 | 56,880 (17.81) | 5,749 (14.74) |
| 3-5 | 76,244 (23.88) | 7,326 (18.78) |
| 6-10 | 76,772 (24.04) | 8,223 (21.08) |
| ≥ 11 | 107,328 (33.61) | 17,460 (44.76) |
| SNF/LTC stay^c^ | 341 (0.11) | 104 (0.27) |
| Influenza vaccination in previous year | 90,018 (28.19) | 12,166 (31.19) |

COVID‑19 = coronavirus disease 2019; ED = emergency department; GBS = Guillain-Barré syndrome; ITP = immune thrombocytopenia; LTC = long-term care; Q1 = first quartile, Q3 = third quartile; SD = standard deviation; SNF = skilled nursing facility; US = United States.

^a^ This serves as an outcome-specific exclusion criterion when the condition is evaluated as an outcome, but history of the condition serves as a covariate for analyses of other outcomes.

^b^ Unique days on which an outpatient visit occurred.

^c^ SNF and LTC both identified in Medicare; SNF identified in MarketScan.

B. Medicare

| Characteristic | COVID-19 diagnosis (exposure) group retained by matching | Individuals with a COVID-19 diagnosis who failed to match |
| --- | --- | --- |
| Total unique individuals | 1,017,410 (1,017,410) | 68,008 (68,008) |
| Demographic characteristics |  |  |
| Age, years |  |  |
| Mean (SD) | 77.7 (8.6) | 80.8 (9.0) |
| Median (Q1, Q3) | 76.0 (71.0, 84.0) | 81.0 (73.0, 88.0) |
| Age groups, N (%) |  |  |
| 65-79 years | 629,709 (61.9) | 31,064 (45.7) |
| ≥ 80 years | 387,701 (38.1) | 36,944 (54.3) |
| Sex, N (%) |  |  |
| Female | 590,584 (58.0) | 34,257 (50.4) |
| Male | 426,826 (42.0) | 33,751 (49.6) |
| US geographic region, N (%) |  |  |
| Northeast | 218,277 (21.5) | 9,103 (13.4) |
| North central | 250,139 (24.6) | 19,879 (29.2) |
| South | 385,012 (37.8) | 31,590 (46.5) |
| West | 161,002 (15.8) | 7,093 (10.4) |
| Unknown | 2,980 (0.3) | 343 (0.5) |
| Race/ethnicity, N (%) |  |  |
| American Indian/Alaska Native | 7,510 (0.7) | 928 (1.4) |
| Asian | 16,819 (1.7) | 904 (1.3) |
| Black or African American | 90,997 (8.9) | 9,789 (14.4) |
| Hispanic/Latin American/Latinx | 26,460 (2.6) | 1,640 (2.4) |
| White | 845,042 (83.1) | 53,588 (78.8) |
| A race/ethnicity not listed | 13,898 (1.4) | 730 (1.1) |
| Not provided | 16,684 (1.6) | 429 (0.6) |
| Dual Medicare/Medicaid eligibility, N (%) | 297,499 (29.2) | 30,409 (44.7) |
| Original reason for Medicare eligibility, N (%) |  |  |
| Age | 860,430 (84.6) | 53,381 (78.5) |
| Disability | 150,916 (14.8) | 14,052 (20.7) |
| End-stage renal disease | 6,064 (0.6) | 575 (0.8) |
| Hospitalized on Time 0, N (%) | 166,154 (16.3) | 67,764 (99.6) |
| SNF/LTC^a^ residence on Time 0, N (%) | 250,193 (24.6) | 33,311 (49.0) |
| Comorbidities, N (%) |  |  |
| Animal exposure/bites or rabies | 3,105 (0.3) | 130 (0.2) |
| Antiphospholipid syndrome | 829 (0.1) | 40 (0.1) |
| Autoimmune disorders | 93,071 (9.1) | 5,722 (8.4) |
| Brain lesions related to secondary narcolepsy | 519 (0.1) | 54 (0.1) |
| Cancer | 272,867 (26.8) | 16,744 (24.6) |
| Chronic lymphocytic leukemia | 6,995 (0.7) | 576 (0.8) |
| Chronic kidney disease or renal disease (other than end-stage renal disease) | 276,654 (27.2) | 29,364 (43.2) |
| End-stage renal disease | 20,229 (2.0) | 2,489 (3.7) |
| Chronic liver disease | 80,781 (7.9) | 5,759 (8.5) |
| Chronic lung diseases | 293,426 (28.8) | 25,836 (38.0) |
| Dementia or other neurologic condition | 350,351 (34.4) | 34,022 (50.0) |
| Diabetes, Type 1 or 2 | 406,448 (39.9) | 33,886 (49.8) |
| Disseminated intravascular coagulation^a^ | 324 (0.0) | 28 (0.0) |
| Heart conditions | 614,855 (60.4) | 49,731 (73.1) |
| Hemiplegia or paraplegia | 61,853 (6.1) | 7,582 (11.1) |
| Herpes simplex infection | 16,959 (1.7) | 948 (1.4) |
| Hypertension | 823,414 (80.9) | 59,938 (88.1) |
| Immunocompromised state | 438,954 (43.1) | 27,919 (41.1) |
| Infection associated with GBS | 51,279 (5.0) | 4,484 (6.6) |
| Viral infection associated with ITP | 3,702 (0.4) | 237 (0.3) |
| Infection associated with myocarditis/pericarditis | 111,923 (11.0) | 11,207 (16.5) |
| Inpatient surgery | 43,070 (4.2) | 5,421 (8.0) |
| Lipid abnormality | 733,659 (72.1) | 48,762 (71.7) |
| Mental health conditions | 441,954 (43.4) | 35,803 (52.6) |
| Nutritional deficiency | 80,039 (7.9) | 9,196 (13.5) |
| Obese or severely obese | 233,833 (23.0) | 16,401 (24.1) |
| Peripheral vascular disease | 318,384 (31.3) | 28,432 (41.8) |
| Pneumonia or lower respiratory tract infection | 237,118 (23.3) | 22,636 (33.3) |
| Sepsis | 59,822 (5.9) | 8,147 (12.0) |
| Sickle cell disease or thalassemia | 2,611 (0.3) | 177 (0.3) |
| Smoking/nicotine dependency | 64,705 (6.4) | 5,848 (8.6) |
| Stroke or cerebrovascular disease | 228,802 (22.5) | 21,214 (31.2) |
| Thromboembolism | 77,562 (7.6) | 7,678 (11.3) |
| Thrombophilia | 11,107 (1.1) | 763 (1.1) |
| Trauma | 24,546 (2.4) | 3,371 (5.0) |
| Tuberculosis | 2,585 (0.3) | 218 (0.3) |
| Healthcare utilization, N (%) |  |  |
| Inpatient stays in previous 365 days |  |  |
| 0 | 729,890 (71.7) | 37,200 (54.7) |
| 1-2 | 204,101 (20.1) | 19,704 (29.0) |
| ≥ 3 | 83,419 (8.2) | 11,104 (16.3) |
| ED visits in previous 365 days |  |  |
| 0 | 572,454 (56.3) | 26,433 (38.9) |
| 1-2 | 282,442 (27.8) | 22,149 (32.6) |
| ≥ 3 | 162,514 (16.0) | 19,426 (28.6) |
| Outpatient provider visits^b^ in previous 365 days |  |  |
| 0 | 93 (0.0) | 13 (0.0) |
| 1-2 | 21,024 (2.1) | 1,189 (1.7) |
| 3-5 | 51,668 (5.1) | 2,598 (3.8) |
| 6-10 | 118,531 (11.7) | 5,352 (7.9) |
| ≥ 11 | 826,094 (81.2) | 58,856 (86.5) |
| SNF/LTC stay | 295,527 (29.0) | 36,920 (54.3) |
| Influenza vaccination in previous year | 570,066 (56.0) | 33,767 (49.7) |

COVID19 = coronavirus disease 2019; ED = emergency department; GBS = Guillain-Barré syndrome; ITP = immune thrombocytopenia; LTC = long-term care; Q1 = first quartile, Q3 = third quartile; SD = standard deviation; SNF = skilled nursing facility; US = United States.

^a^ This serves as an outcome-specific exclusion criterion when the condition is evaluated as an outcome, but history of the condition serves as a covariate for analyses of other outcomes.

^b^ Unique days on which an outpatient visit occurred.
