# Supplementary material for: Risk of neurologic or immune-mediated adverse events after COVID-19 diagnosis in the United States
Source: PLoS One. 2025 Nov 24;20(11):e0333704. doi: 10.1371/journal.pone.0333704 (PMC12643290; doi:10.1371/journal.pone.0333704)
Supplement: S7 Fig — (DOCX) [file pone.0333704.s007.docx]

S7 Fig. Balance of Covariate Distributions Among Individuals With a COVID‑19 Diagnosis and Comparator Individuals Without a COVID‑19 Diagnosis, Cohort Design, Before and After Stabilized Inverse Probability of Treatment Weighting

A. MarketScan

i. Guillain-Barré Syndrome


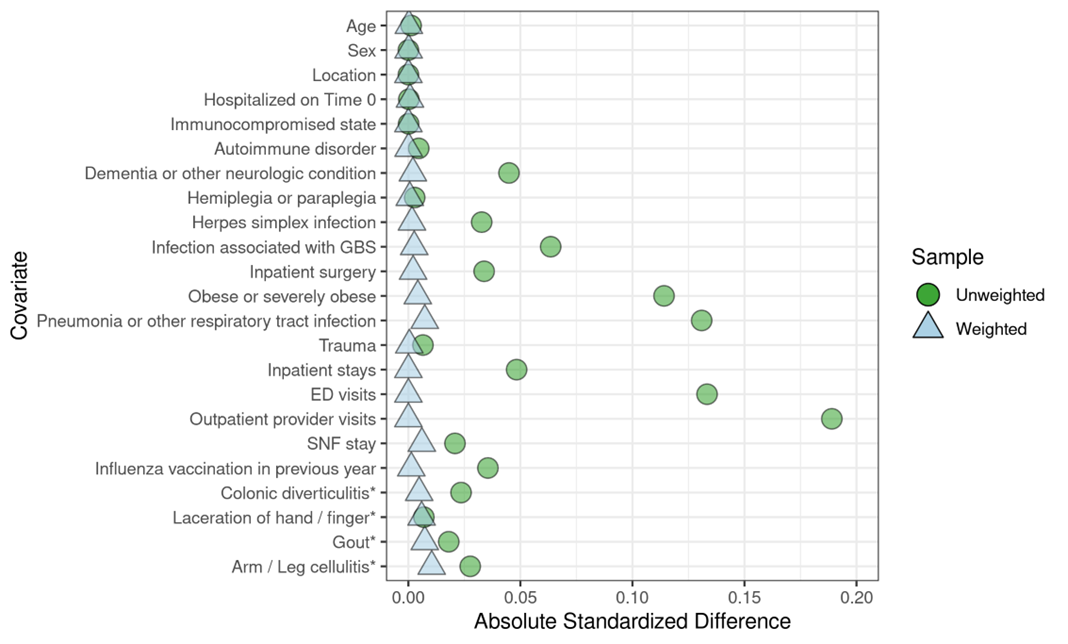


ii. Bell’s Palsy


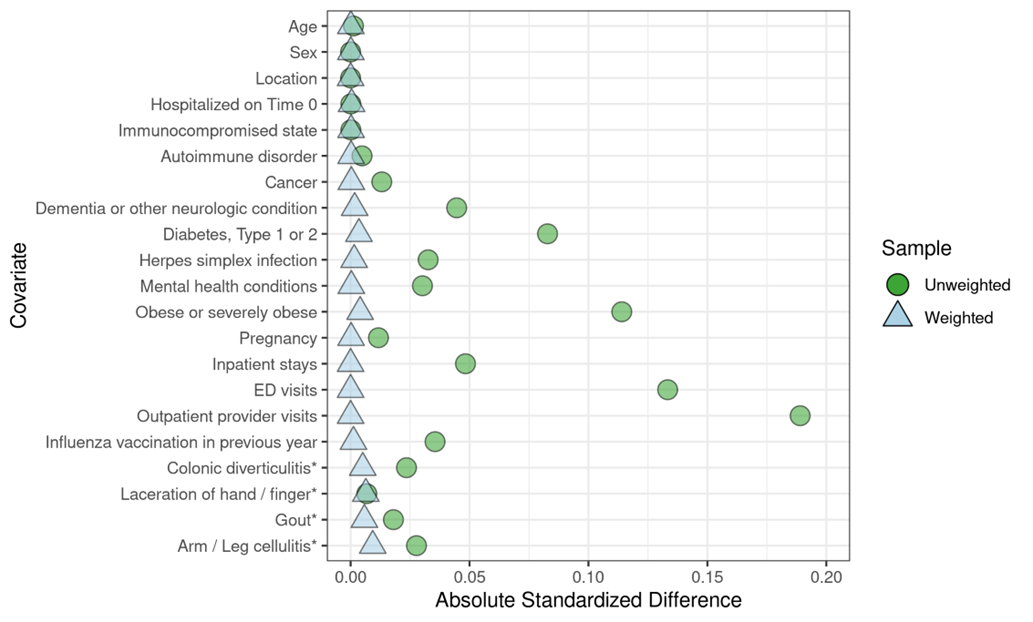


iii. Narcolepsy


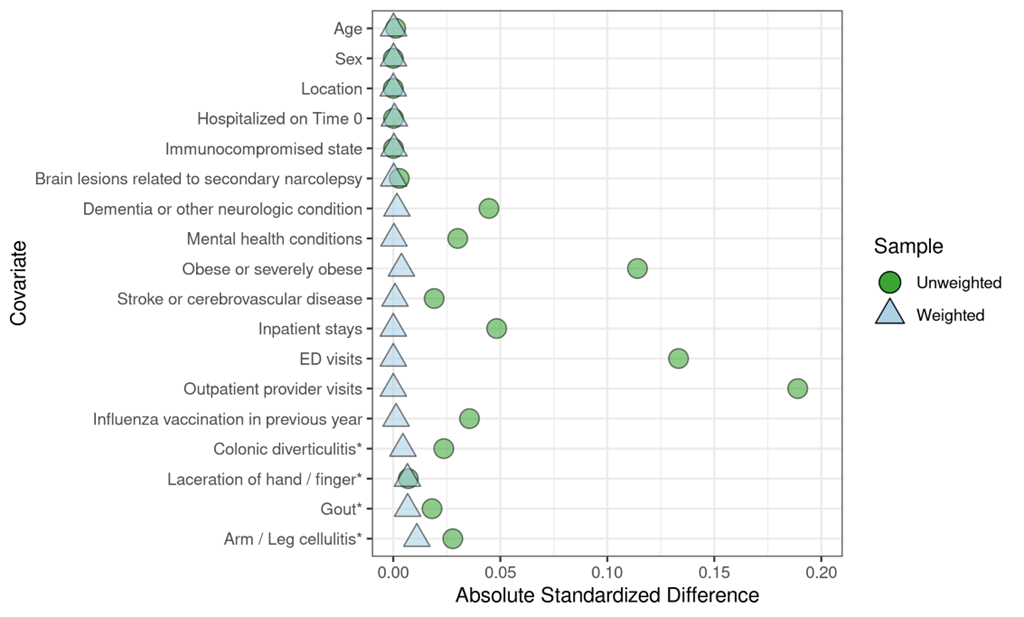


iv. Immune Thrombocytopenia


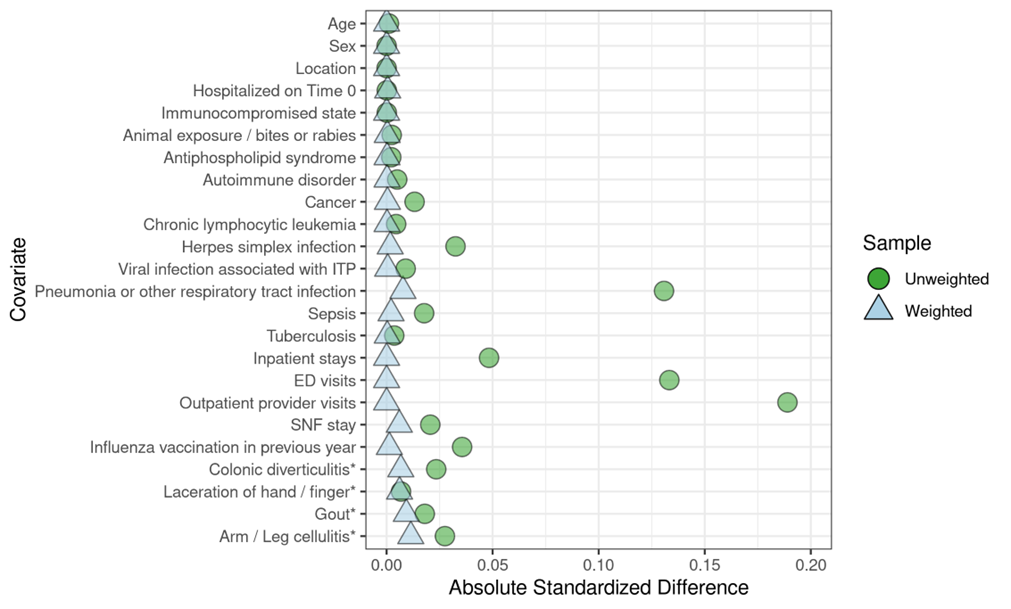


v. Transverse Myelitis


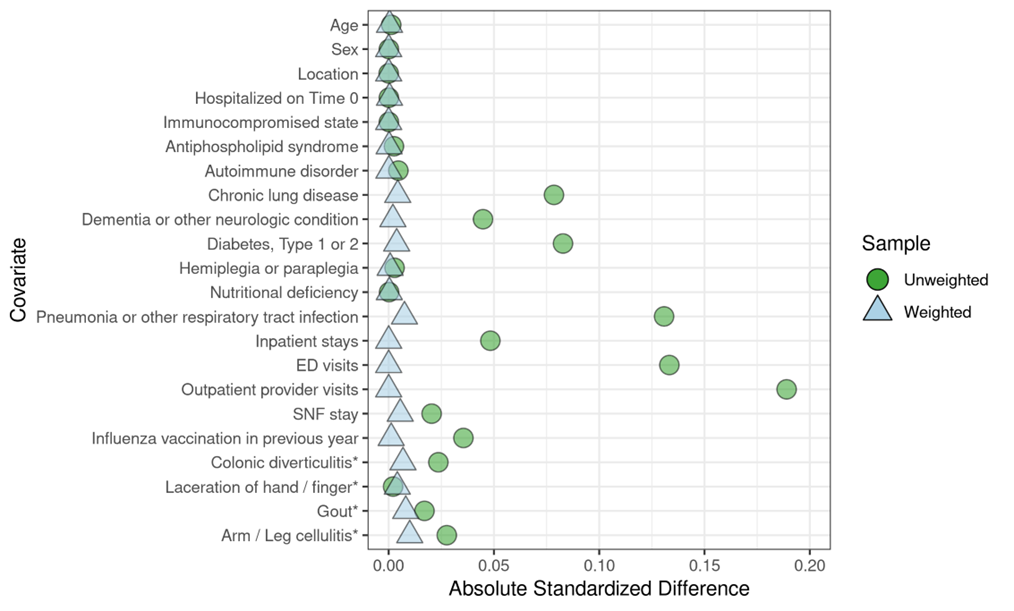


B. Medicare

i. Guillain-Barré Syndrome


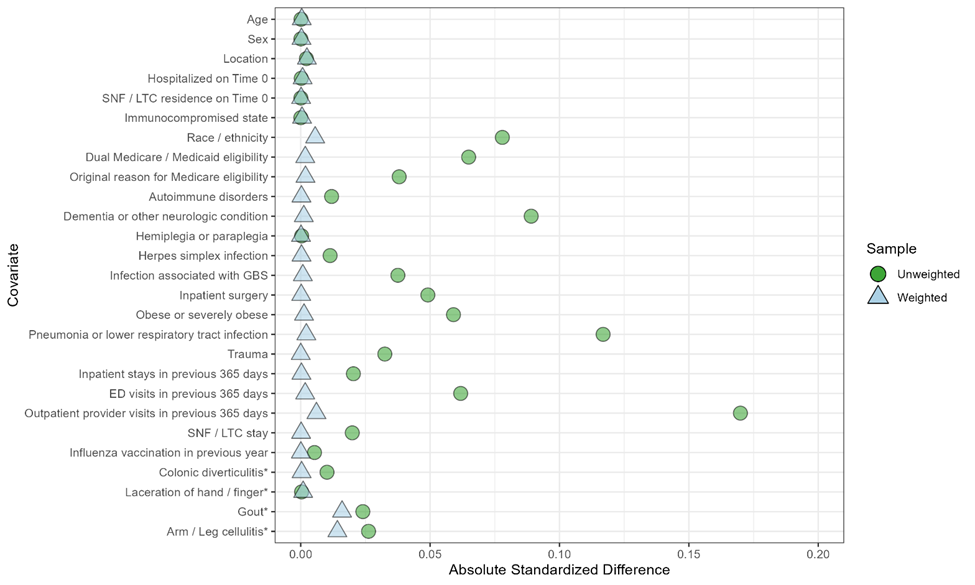


ii. Bell’s Palsy


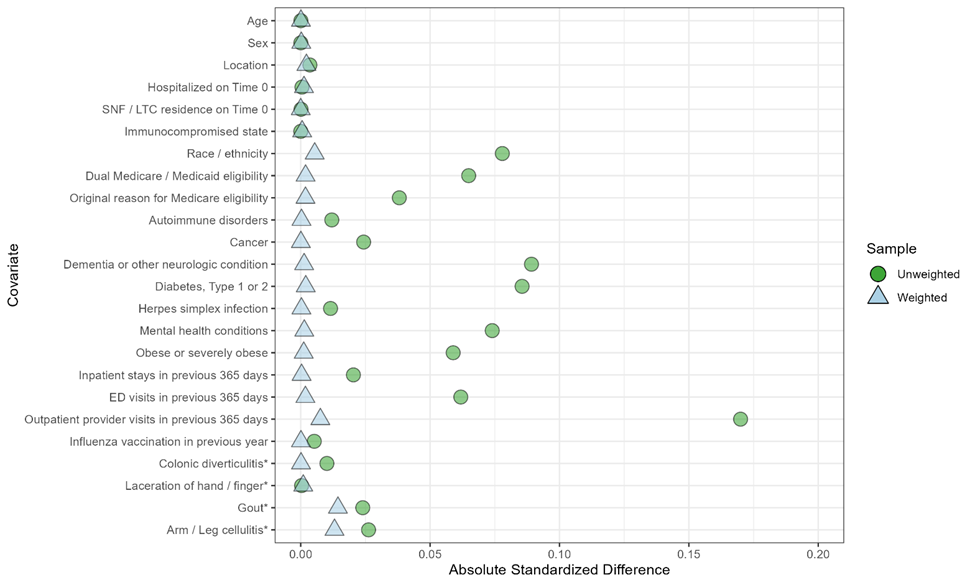


iii. Narcolepsy


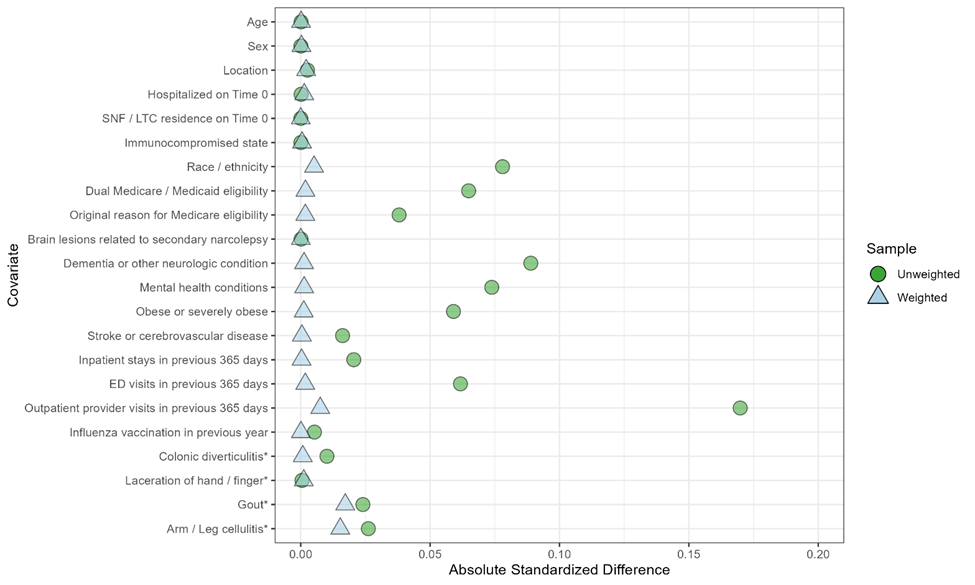


iv. Immune Thrombocytopenia


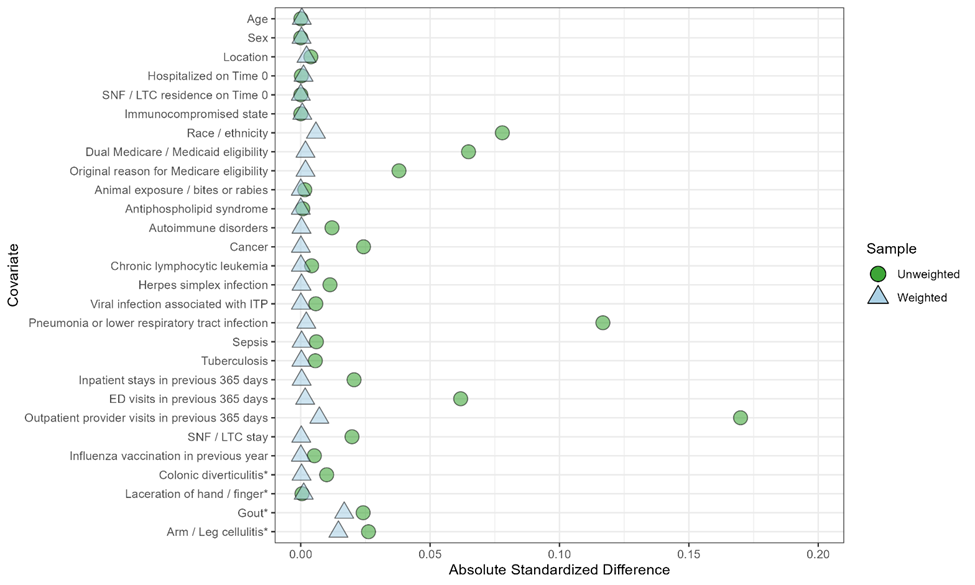


v. Transverse Myelitis


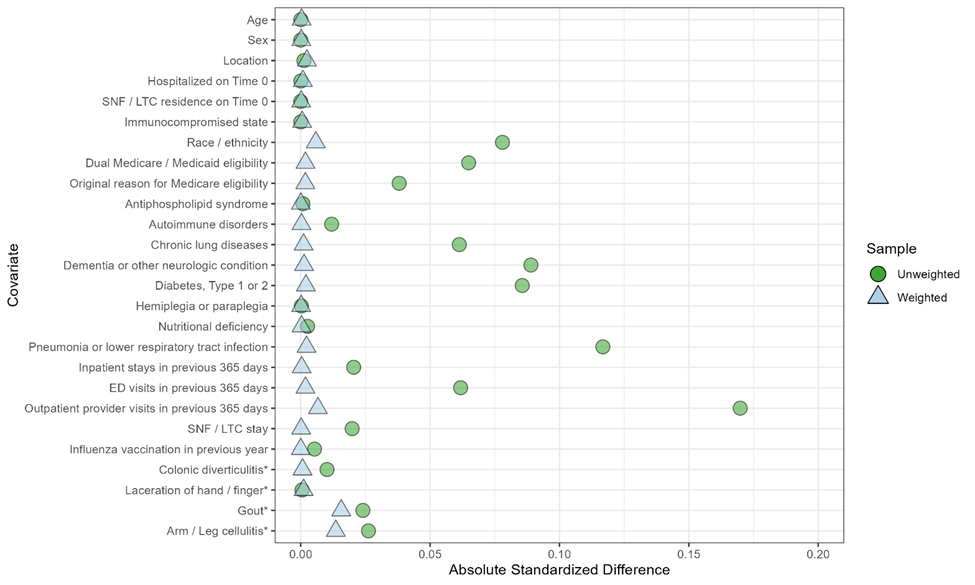


COVID 19 = coronavirus disease 2019; ED = emergency department; GBS = Guillain-Barré syndrome; ITP = immune thrombocytopenia; LTC = long-term care; SNF = skilled nursing facility.
