# Supplementary material for: Risk of neurologic or immune-mediated adverse events after COVID-19 diagnosis in the United States
Source: PLoS One. 2025 Nov 24;20(11):e0333704. doi: 10.1371/journal.pone.0333704 (PMC12643290; doi:10.1371/journal.pone.0333704)
Supplement: S8 Table — (DOCX) [file pone.0333704.s008.docx]

S8 Table. Estimated Attributable Risk of Adverse Events Associated With COVID-19 Diagnoses

| Adverse event | MarketScan | | Medicare | |
| --- | --- | --- | --- | --- |
|  | RI (95% CI) ^a^ | AR per 100,000 COVID-19 cases | RI (95% CI) ^a^ | AR per 100,000 COVID-19 cases |
| Guillain-Barré syndrome | 8.53 (2.45-29.65) | 1.33 | 4.63 (1.78-12.01) | 1.56 |
| Bell’s palsy | 1.95 (1.39-2.74) | 6.92 | 1.05 (0.91-1.22) | 1.50 |
| Narcolepsy | 1.44 (0.84-2.47) | 1.57 | 1.15 (0.85-1.55) | 1.01 |
| Immune thrombocytopenia | 1.74 (1.01-3.00) | 2.31 | 1.91 (1.60-2.28) | 13.98 |
| Transverse myelitis | 3.14 (0.56-17.50) | 0.41 | 0 (0-NE) | 0 |

AR = attributable risk; CI = confidence interval; COVID-19 = coronavirus disease 2019; NE = not estimable; RI = relative incidence.

^a^ Attributable risk estimates derived from the self-controlled risk interval analyses starting follow-up the day after Time 0 with the extended model accounting for event-dependent observation windows and adjusted for seasonality.
