# Supplementary material for: Risk of neurologic or immune-mediated adverse events after COVID-19 diagnosis in the United States
Source: PLoS One. 2025 Nov 24;20(11):e0333704. doi: 10.1371/journal.pone.0333704 (PMC12643290; doi:10.1371/journal.pone.0333704)
Supplement: S9 Table — (DOCX) [file pone.0333704.s009.docx]

S9 Table. Association of a COVID-19 Diagnosis With Adverse Events, Cohort Design, Follow-Up Starting on Time 0

| Adverse event | Data source | Exposure group | Outcome cases | Person-time (days) | Crude cumulative incidence^a^ (95% CI) | Crude HR (95% CI) | sIPT-weighted HR (95% CI) |
| --- | --- | --- | --- | --- | --- | --- | --- |
| Guillain-Barré syndrome | MarketScan | COVID-19 | 11 | 26,740,944 | 5.44 (2.07-8.80) | 10.99 (1.42-85.10) | 10.69 (1.38-82.74) |
|  |  | Comparator | 1 | 26,735,597 | 1.48 (0.00-4.37) | — | — |
|  | Medicare | COVID-19 | 45 | 81,022,327 | 6.73 (4.58-8.89) | 1.03 (0.66-1.62) | 1.06 (0.67-1.68) |
|  |  | Comparator | 45 | 87,266,038 | 16.41 (0.00-37.81) | — | — |
| Bell’s palsy | MarketScan | COVID-19 | 122 | 26,710,504 | 116.84 (76.12-157.53) | 1.31 (1.00-1.73) | 1.22 (0.92-1.61) |
|  |  | Comparator | 93 | 26,715,069 | 70.60 (51.68-89.51) | — | — |
|  | Medicare | COVID-19 | 976 | 80,836,700 | 229.91 (208.74-251.07) | 1.12 (1.01-1.23) | 1.06 (0.96-1.17) |
|  |  | Comparator | 923 | 87,065,654 | 200.94 (178.86-223.02) | — | — |
| Encephalitis/ encephalomyelitis | MarketScan | COVID-19 | 11 | 26,738,915 | 5.28 (1.99-8.58) | 2.20 (0.76-6.33) | 1.99 (0.69-5.77) |
|  |  | Comparator | 5 | 26,737,424 | 6.17 (0.00-13.19) | — | — |
|  | Medicare | COVID-19 | 193 | 81,037,202 | 35.70 (27.29-44.1) | 1.44 (1.12-1.85) | 1.43 (1.10-1.85) |
|  |  | Comparator | 139 | 87,288,050 | 22.15 (17.02-27.28) | — | — |
| Narcolepsy | MarketScan | COVID-19 | 36 | 26,713,511 | 40.71 (20.30-61.12) | 1.29 (0.78-2.13) | 1.17 (0.70-1.94) |
|  |  | Comparator | 28 | 26,712,922 | 26.57 (11.61-41.53) | — | — |
|  | Medicare | COVID-19 | 218 | 80,972,196 | 49.87 (39.28-60.46) | 1.32 (1.07-1.64) | 1.22 (0.99-1.52) |
|  |  | Comparator | 174 | 87,225,920 | 52.25 (18.60-85.90) | — | — |
| Immune thrombocytopenia | MarketScan | COVID-19 | 47 | 26,717,161 | 29.14 (19.46-38.83) | 2.14 (1.29-3.54) | 2.10 (1.26-3.51) |
|  |  | Comparator | 22 | 26,720,033 | 17.17 (8.34-26.00) | — | — |
|  | Medicare | COVID-19 | 693 | 80,830,449 | 127.49 (114.63-140.36) | 1.46 (1.29-1.65) | 1.43 (1.27-1.62) |
|  |  | Comparator | 496 | 87,072,175 | 115.09 (90.40-139.77) | — | — |
| Transverse myelitis | MarketScan | COVID-19 | 3 | 26,740,061 | 0.99 (0.00-2.11) | 1.50 (0.25-8.97) | 1.70 (0.28-10.19) |
|  |  | Comparator | 2 | 26,737,142 | 0.87 (0.00-2.12) | — | — |
|  | Medicare | COVID-19 | < 11 | 81,061,561 | 1.54 (0.45-2.64) | 0.54 (0.23-1.29) | 0.59 (0.25-1.43) |
|  |  | Comparator | 17 | 87,313,779 | 2.22 (0.95-3.50) | — | — |

CI = confidence interval; COVID‑19 = coronavirus disease 2019; HR = hazard ratio; sIPT = stabilized inverse probability of treatment.

— denotes the reference group.

^a^ Estimated as the 1 minus the Kaplan-Meier survival estimator; expressed as risk per 100,000 individuals.

Note: Privacy rules for Medicare require masking cell sizes of fewer than 11 individuals.
