# Supplementary material for: Risk of neurologic or immune-mediated adverse events after COVID-19 diagnosis in the United States
Source: PLoS One. 2025 Nov 24;20(11):e0333704. doi: 10.1371/journal.pone.0333704 (PMC12643290; doi:10.1371/journal.pone.0333704)
Supplement: S10 Table — (DOCX) [file pone.0333704.s010.docx]

S10 Table. Association of a COVID-19 Diagnosis With Adverse Events, SCRI Design, MarketScan and Medicare, Follow-Up Starting on Time 0

| Adverse event | Outcomes | Cases in risk window | Cases in reference window | RI (95% CI) ^a^ |
| --- | --- | --- | --- | --- |
| Guillain-Barré syndrome | MarketScan | 7 | 5 | 14.30 (2.34-87.19) |
|  | Medicare | 29 | 18 | 11.65 (4.28-31.69) |
| Bell’s palsy | MarketScan | 62 | 130 | 2.60 (1.83-3.70) |
|  | Medicare | 450 | 1,030 | 1.94 (1.71-2.21) |
| Narcolepsy | MarketScan | 22 | 64 | 1.87 (1.08-3.26) |
|  | Medicare | 113 | 225 | 2.05 (1.58-2.66) |
| Immune thrombocytopenia | MarketScan | 29 | 49 | 3.03 (1.74-5.28) |
|  | Medicare | 451 | 469 | 3.86 (3.28-4.54) |
| Transverse myelitis | MarketScan | 3 | 4 | 4.84 (0.78-30.11) |
|  | Medicare | < 11 | > 11 | 3.76 (1.25-11.35) |

CI = confidence interval; COVID 19 = coronavirus disease 2019; RI = relative incidence; SCRI = self-controlled risk interval.

^a^ Conditional Poisson model accounting for event-dependent observation windows and adjusted for calendar month, with December treated as a full calendar month.

Note: Privacy rules for Medicare require masking cell sizes of fewer than 11 individuals.
